# Pharmacological Protein Kinase C Modulators Reveal a Pro-hypertrophic Role for Novel Protein Kinase C Isoforms in Human Induced Pluripotent Stem Cell-Derived Cardiomyocytes

**DOI:** 10.3389/fphar.2020.553852

**Published:** 2021-01-20

**Authors:** Lotta Pohjolainen, Julia Easton, Reesha Solanki, Heikki Ruskoaho, Virpi Talman

**Affiliations:** Drug Research Program and Division of Pharmacology and Pharmacotherapy, Faculty of Pharmacy, University of Helsinki, Helsinki, Finland

**Keywords:** cardiomyocyte hypertrophy, hiPSC-derived cardiomyocyte, neonatal rat ventricular cardiomyocyte, endothelin-1, protein kinase C, high content analysis, protein kinase C agonists

## Abstract

**Background:** Hypertrophy of cardiomyocytes (CMs) is initially a compensatory mechanism to cardiac overload, but when prolonged, it leads to maladaptive myocardial remodeling, impairing cardiac function and causing heart failure. A key signaling molecule involved in cardiac hypertrophy is protein kinase C (PKC). However, the role of different PKC isoforms in mediating the hypertrophic response remains controversial. Both classical (cPKC) and novel (nPKC) isoforms have been suggested to play a critical role in rodents, whereas the role of PKC in hypertrophy of human CMs remains to be determined. Here, we aimed to investigate the effects of two different types of PKC activators, the isophthalate derivative HMI-1b11 and bryostatin-1, on CM hypertrophy and to elucidate the role of cPKCs and nPKCs in endothelin-1 (ET-1)-induced hypertrophy *in vitro*.

**Methods and Results:** We used neonatal rat ventricular myocytes (NRVMs) and human induced pluripotent stem cell-derived cardiomyocytes (hiPSC-CMs) to study the effects of pharmacological PKC modulators and ET-1. We used quantitative reverse transcription PCR to quantify hypertrophic gene expression and high-content analysis (HCA) to investigate CM morphology. In both cell types, ET-1, PKC activation (bryostatin-1 and HMI-1b11) and inhibition of cPKCs (Gö6976) increased hypertrophic gene expression. In NRVMs, these treatments also induced a hypertrophic phenotype as measured by increased recognition, intensity and area of α-actinin and F-actin fibers. Inhibition of all PKC isoforms with Gö6983 inhibited PKC agonist-induced hypertrophy, but could not fully block ET-1-induced hypertrophy. The mitogen-activated kinase kinase 1/2 inhibitor U0126 inhibited PKC agonist-induced hypertrophy fully and ET-1-induced hypertrophy partially. While ET-1 induced a clear increase in the percentage of pro-B-type natriuretic peptide-positive hiPSC-CMs, none of the phenotypic parameters used in HCA directly correlated with gene expression changes or with phenotypic changes observed in NRVMs.

**Conclusion:** This work shows similar hypertrophic responses to PKC modulators in NRVMs and hiPSC-CMs. Pharmacological PKC activation induces CM hypertrophy via activation of novel PKC isoforms. This pro-hypertrophic effect of PKC activators should be considered when developing PKC-targeted compounds for e.g. cancer or Alzheimer’s disease. Furthermore, this study provides further evidence on distinct PKC-independent mechanisms of ET-1-induced hypertrophy both in NRVMs and hiPSC-CMs.

## Introduction

Cardiovascular diseases are the most common causes of death worldwide, accounting for almost one third of all deaths (GBD 2015 Mortality and Causes of Death Collaborators, 2016). Heart failure, caused most often by a myocardial infarction, hypertension and cardiomyopathies, affects more than 23 million people globally (Bui et al., 2011). Despite the recent advances in therapies, heart failure has a poor prognosis as half of the patients die within 5 years after diagnosis. The heart adapts to cardiac overload by excessive growth, i.e. hypertrophy of individual muscle cells, which is characterized by increased protein synthesis, addition and reorganization of sarcomeres and changes in gene expression, such as upregulation of natriuretic peptide A and B coding genes *NPPA* and *NPPB*, respectively (Heineke and Molkentin, 2006; van Berlo et al., 2013). Cardiac hypertrophy is initially a compensatory response to normalize the increased pressure to the left ventricular walls. However, when prolonged it leads to maladaptive myocardial remodeling, which impairs cardiac function and can eventually lead to heart failure. As left ventricular hypertrophy is a key risk factor for the development of heart failure, several molecular pathways are under investigation for the potential ability to modulate hypertrophic growth (Bernardo et al., 2010; van Berlo et al., 2013; Tham et al., 2015).

One key regulator of cardiomyocyte hypertrophy is protein kinase C (PKC) (Singh et al., 2017). PKC is a serine/threonine kinase family consisting of 10 isoforms (Naruse and King, 2000). By phosphorylating serine and threonine residues of numerous protein targets, they function as important mediators of many signaling pathways and contribute to various cellular processes, including growth, differentiation, proliferation and tumorigenesis. PKCs are divided into three groups based on their structure and activation: classical (cPKC; α, βI, βII and γ), novel (nPKC; δ, ε, η and *θ*) and atypical (aPKC; ζ and ι/λ) isoforms. The cPKCs are activated by Ca^2+^, diacylglycerol (DAG) and phosphatidylserine (PS), whereas the nPKCs are activated by DAG and PS independent of Ca^2+^. In contrast, the aPKCs are activated independently of Ca^2+^, DAG and PS. Activation of cPKCs and nPKCs involves translocation of the enzyme from the cytosol to the cell membrane, where the second messenger DAG binds to the so-called C1 domain in the regulatory region of PKC, which in turn induces a conformational change that leads to activation (Steinberg, 2008). The C1 domain is also the binding site for naturally occurring ultrapotent PKC activators such as phorbol esters and bryostatins (Boije af Gennäs et al., 2011). Although the same ligands activate many isoforms, not all isoforms are activated upon same stimuli. Isoform-specific activation is suggested to occur through interaction of PKC with isoform-selective receptors for activated C kinase (RACKs) (Schechtman and Mochly-Rosen, 2001). These membrane-associated proteins aid individual PKC isoforms to localize uniquely.

In addition to isoform-selective RACKs, PKC isoforms have species-, tissue-, and age-specific expression patterns (Singh et al., 2017). In rat cardiomyocytes (CMs), isoforms α, βI, βII, δ, ε, ζ and η have been identified, while in human hearts, all isoforms except γ and *θ* have been described (Kohout and Rogers, 1993; Rybin and Steinberg, 1994; Clerk et al., 1995; Shin et al., 2000; Simonis et al., 2007). Based on differential expression and activation in hypertrophied and failed hearts and in *in vitro* experiments with CMs, particularly isoforms α, β, δ, and ε have been suggested to mediate CM hypertrophy (Singh et al., 2017). In an attempt to reveal the roles of individual isoforms, different approaches have been utilized, such as genetic modulation by transgenic overexpression (Shubeita et al., 1992; Bowman et al., 1997; Wakasaki et al., 1997; Takeishi et al., 2000; Braz et al., 2002) or gene ablation (Roman et al., 2001; Braz et al., 2004; Gray et al., 2004; Klein et al., 2005; Liu et al., 2009; Song et al., 2015), and modulation of PKC isoform translocation using RACK binding or pseudo-RACK peptides (Mochly-Rosen et al., 2000; Chen et al., 2001; Stebbins and Mochly-Rosen, 2001; Hahn et al., 2003). The results of these studies have, however, been conflicting, which might be explained by cross-regulation and redundancy of PKC isoforms.

PKC has been considered an attractive drug target for various diseases, including heart diseases, cancer, autoimmune diseases and central nervous system diseases (Mochly-Rosen et al., 2012). Cancer, in particular, has been subject of intensive research. However, drug development has struggled with designing isoform-specific and selective PKC activators and inhibitors. In addition to the difficulties in drug design and synthesis, understanding that downregulation of PKC protein and thus decreased PKC activity upon prolonged strong PKC activation is actually responsible for the tumor-promoting effects of ultrapotent PKC agonists has only recently led to the reversal of PKC-related dogma: for the treatment of cancer, PKC activators that do not cause downregulation should be developed instead of inhibitors (Antal et al., 2015; Newton and Brognard, 2017). Indeed, only one PKC-targeted drug has been approved until now: the C1 domain-binding PKC activator ingenol mebutate was approved for the treatment of actinic keratosis, precursor of squamous cell carcinoma in 2012, but has recently been withdrawn from the market due to risk of skin cancer (Freiberger et al., 2015; European Medicines Agency, 2020). In addition, another PKC activator, tigilanol tiglate, developed for treatment of broad range of tumors, has showed promising results in Phase I study (Panizza et al., 2019). The nature-derived macrocyclic lactone bryostatin-1 has also been investigated in several clinical trials against cancer, Alzheimer’s disease and HIV (Wu et al., 2020). Dialkyl 5-(hydroxymethyl) isophtalates (HMIs) are PKC activators that bind to the C1 domains of cPKCs and nPKCs at low micromolar concentrations (Boije af Gennäs et al., 2009). While the isophthalates, such as HMI-1b11, activate PKC in cellular context and induce PKC-dependent extracellular signal-regulated kinase 1/2 (ERK1/2) phosphorylation, they seem to act as partial agonists instead of full activators (Boije af Gennäs et al., 2009; Talman et al., 2011; Talman et al., 2013). Together with their easy synthesis, partial agonism can be considered an advantage for the isophthalates, as they do not induce PKC downregulation (Sarajärvi et al., 2018) and thus more closely mimic physiological activation by DAG.

As PKC activators are being developed for the treatment of many diseases, it is highly important to characterize their cardiac effects. The aim of this study was to examine the effects of two different types of PKC activators, isophthalates and bryostatins, on CM hypertrophy. As a large part of the research regarding PKC is based on genetic studies done in mice and most of the hypertrophy studies have been done in rodents, they may not be directly applicable to human, and it therefore is important to compare rodent and human models. Human induced pluripotent stem cell-derived cardiomyocytes (hiPSC-CMs) enable investigation of human CMs and provide a unique platform to model genetic cardiomyopathies (Karakikes et al., 2015; Yoshida and Yamanaka, 2017; Brodehl et al., 2019). To compare human and rodent CMs, we elucidated the role of cPKC and nPKC isoforms in endothelin-1 (ET-1)-induced hypertrophy *in vitro* both in neonatal rat ventricular myocytes (NRVMs) and in hiPSC-CMs.

## Materials and Methods

### Compounds and Reagents

The PKC activator HMI-1b11 (bis (1-ethylpentyl) 5-(hydroxymethyl) isophthalate) was synthesized at the Division of Pharmaceutical Chemistry and Technology, Faculty of Pharmacy, University of Helsinki (Finland), as described previously (Boije af Gennäs et al., 2009) and used at 10 μM concentration previously shown to bind and activate PKC (Boije af Gennäs et al., 2009; Talman et al., 2013). The PKC activator bryostatin-1 was purchased from Sigma-Aldrich (Steinheim, Germany), the cPKC inhibitor Gö6976 from Merck Millipore (Burlington, MA, United States), the pan-PKC inhibitor Gö6983 from Stemcell Technologies (Vancouver, Canada), and the mitogen-activated protein kinase kinase 1/2 (MEK1/2) inhibitor U0126 from Tocris Bioscience (Bristol, United Kingdom). The concentrations of the commercially available PKC and MEK1/2 modulators were chosen based on reported *K*_i_ values and our previous work (Boije af Gennäs et al., 2009; Talman et al., 2013; Sarajärvi et al., 2018). Endothelin-1 (ET-1), purchased from Sigma-Aldrich, was dissolved in 1% bovine serum albumin (BSA) in Dulbecco’s modified Eagle medium (DMEM), while all other compounds were dissolved in dimethyl sulfoxide (DMSO). The ET-1 concentration of 100 nM was used to stimulate hypertrophy, as described earlier (Välimäki et al., 2017).

Cell culture reagents were purchased from Gibco (Paisley, United Kingdom) unless otherwise stated. Collagenase type 2 was purchased from Worthington Biochemical Corporation (Lakewood, NJ, United States). BSA (A4919), DMEM, insulin–transferrin–sodium selenite media supplement (I1884), pancreatin (P-3292), sodium pyruvate, 3,3′,5-triiodo-L-thyronine (T_3_) and all reagents used in cytotoxicity assays were purchased from Sigma-Aldrich. Growth Factor-Reduced Matrigel was bought from Corning (Bedford, MA, United States), and small-molecule inhibitors Y-27632, CHIR99021 and Wnt-C59 used in hiPSC culture and differentiation were from Tocris Bioscience (Bristol, United Kingdom). Ultrapure gelatin was acquired from Merck Millipore.

### Cell Culture

Neonatal rat ventricular myocyte (NRVM) cultures were prepared from 1–3 days old Wistar rats (both sexes) as described previously (Välimäki et al., 2017; Karhu et al., 2018). Animals were housed and terminated in accordance with the 3R principles of the EU directive 2010/63/EU governing the care and use of experimental animals and following local laws and regulations. Rats were sacrificed by decapitation and ventricles were dissected, cut into 5–8 pieces and digested mechanically and enzymatically by shaking at 600 rpm at 37°C in a digestion buffer containing 2 mg/ml collagenase type 2, 2 mg/ml pancreatin, 100 mM NaCl, 10 mM KCl, 1.2 mM KH_2_PO_4_, 4 mM MgSO_4_·6H_2_O, 50 mM taurine, 20 mM glucose, 100 U/ml penicillin, 100 µg/ml streptomycin and 10 mM 4-(2-hydroxyethyl)-1-piperazine ethanesulfonic acid (HEPES; pH 6.9) for 1–2 h. The cell suspension was collected and centrifuged at 160 g for 5 min, whereafter the pellet containing live cardiac cells was suspended in DMEM/F12 medium containing 10% fetal bovine serum (FBS), 100 U/ml penicillin and 100 µg/ml streptomycin. The cells were pre-plated into cell culturing flasks and incubated at 37°C in a humidified atmosphere of 5% CO_2_ for 1–2 h to let non-myocytes attach. The enriched, unattached CMs were collected with the medium and seeded on 96-well plates at 40,000 cells/well for cytotoxicity assays or at 30,000–40,000 cells/well for immunofluorescence staining. For quantitative reverse transcription PCR (qRT-PCR), the cells were plated on 12-well plates at 500,000 cells/well. For Western blotting, the cells were plated on 6-well plates at 10^6^ cells/well. The plating medium was changed to complete serum free medium (CSFM; DMEM/F12 containing 2.5 mg/ml BSA, 100 units/ml penicillin, 100 µg/ml streptomycin, 5 µg/ml insulin, 5 μg/ml transferrin, 5 ng/ml selenium, 2.8 mM sodium pyruvate, and 0.1 nM T_3_) on the following day, whereafter the cells were let to adapt for 24 h before pharmacological treatments. All pharmacological treatments were done using CSFM.

HiPSC-CMs were produced from iPS (IMR90)-4 line (WiCell, Madison, Wisconsin, United States) using a differentiation protocol described previously (Burridge et al., 2014; Karhu et al., 2018). Briefly, the hiPSC cultures were maintained in Essential eight™ medium (E8) on Matrigel (1:50)-coated 6-well plates and passaged 1:15 approximately every four days using EDTA. When the cultures were 80–95% confluent, differentiation was started by adding 6 µM CHIR99021 (day 0) in RPMI 1640 medium supplemented with B-27 without insulin (RB-). After 24 h (day 1), the medium was changed to fresh RB- without CHIR99021. On day 3, fresh RB- containing 2.5 µM C59 was added for 48 h. The cells were fed with RB- on days 5, 7 and 9. On day 11, metabolic selection of CMs was initiated by changing RB- to RPMI 1640 without glucose supplemented with B-27 (with insulin). After 48 h, the cells were fed with fresh metabolic selection medium. From day 15 onwards, the cardiomyocytes were cultured in RPMI 1640 supplemented with B-27 (RB+). On day 15 or 17, the differentiated hiPSC-CMs were dissociated and seeded on gelatin-coated 96-well plates at 17,500–25,000 cells/well for cytotoxicity assays and immunofluorescence staining or on gelatin-coated 12-well plates at 480,000–500,000 cells/well for qRT-PCR in RB+ supplemented with 10% FBS. For Western blotting, the cells were seeded on 6-well plates at 700,000 cells/well. After letting the cells attach at least for 48 h, the medium was changed to serum-free RB+ or treated with test compounds for cytotoxicity assays or Western blotting. For immunofluorescence staining and qRT-PCR, the hiPSC-CMs were maintained at least until day 30 before pharmacological treatments. All treatments were done using RB+. The differentiation protocol produces 0.5–1.6 × 10^6^ CMs per one well of a 6-well plate. Only differentiations with >95% pure cardiomyocytes as assessed by visual inspection of beating cells on differentiation plates prior to detachment were used. Furthermore, staining for α-actinin and/or cardiac troponin T were used to confirm the purity of the CM population (>95% at the time of imaging). All experiments with hiPSCs were carried out in accordance with the Finnish Act on the Medical Use of Human Organs, Tissues and Cells (101/2001). No human tissue samples were collected for the study. All cell cultures were maintained at 37°C in a humidified atmosphere of 5% CO_2_.

### Cytotoxicity Assays

The cytotoxicity of the compounds was studied using lactate dehydrogenase (LDH) assay to determine cell membrane integrity and 3-(4,5-dimethyl-2-thiazolyl)-2,5-diphenyltetrazolium bromide (MTT) assay to measure mitochondrial metabolic activity, as described previously (Talman et al., 2011). For cytotoxicity assays, the cells were exposed to compounds at different concentrations or to vehicle (0.3% DMSO) for 24 h.

To measure cell membrane integrity, 50 μl of the medium from each well was transferred to a new 96-well plate and 50 μl of substrate solution containing 1.3 mM β-nicotinamide adenine nucleotide, 660 μM iodonitrotetrazolium, 54 mM L (+)-lactic acid, 280 μM phenazine methosulphate and 0.2 M Tris-HCl (pH 8.0) was added. After a 30-min incubation at room temperature (RT) with shaking at 400 rpm for the first 10 min, formazan formation was stopped by adding 50 μl of 1 M acetic acid to each well. Absorbance at 490 nm was measured with Victor2 1420 Multilabel Counter (PerkinElmer, Waltham, MA, United States). To calculate the cytotoxicity, background absorbance measured from wells with medium but no cells was subtracted from all values, after which the spontaneous LDH release from untreated cells was subtracted and the values were compared to maximal LDH release of cells lysed with 0.9% Triton X-100.

To measure the metabolic activity, MTT was added to the cells at a final concentration of 0.5 mg/ml. After a 2-h incubation at 37°C in a humidified atmosphere of 5% CO_2_, the medium was removed, and the formazan crystals were dissolved in 200 μl of DMSO per well. Absorbance was measured with Bio-Rad plate reader (Hercules, CA, United States) at 550 nm subtracting the absorbance at 650 nm as background. The cell viability was calculated by comparing the absorbance values to the absorbance of untreated wells. In each independent experiment, at least two technical replicates were used for each treatment and the average of the technical replicates was used for analysis.

### Protein Sample Preparation, SDS-PAGE and Western Blotting

For analysis of PKC isoform expression, CMs on gelatin-coated 6-well plates were exposed to PKC agonists for 48 h. For analysis of ERK1/2 phosphorylation, CMs on gelatin-coated 6-well plates were exposed to PKC inhibitors or MEK1/2 inhibitor for 10–15 min before adding PKC agonists for 30 min. CMs were then washed two times with PBS and lysed in 150 µl of 1% sodium dodecyl sulphate (SDS) in 50 mM Tris-HCl (pH 7.5) and genomic DNA was sheared either by sonication or passing the lysate through a 25-gauge needle repeatedly. The protein concentration was measured using the Pierce BCA protein assay kit (Thermo Scientific) according to manufacturer’s instructions. Samples were diluted in Laemmli sample buffer at equal concentrations, boiled for 5 min and stored at ‒20°C until use. Protein samples (equal amount of protein per lane, 10–25 µg depending on the experiment) were separated using SDS-PAGE and subsequently transferred to nitrocellulose membranes. Membranes were washed with 0.1% Tween 20 in Tris-buffered saline (TTBS) for 5 min, blocked with 5% nonfat milk powder in TTBS (milk-TTBS) for 1 h, and incubated with primary antibodies in milk-TTBS at 4°C overnight. The following primary antibody dilutions were used: anti-PKCα (ab32376, Abcam; 1:1,000), anti-PKCβI (ab195039, Abcam; 1:2,000), anti-PKCβII (ab32026, Abcam; 1:1,000), anti-PKC-δ (ab182126, Abcam; 1:5,000); anti-PKCε (ab124806, Abcam; 1:1,000), anti-PKCη (ab179524, Abcam; 1:2,000), anti-ERK1/2 (sc-94, Santa Cruz; 1:1,000), anti-phospho-ERK1/2 (9101, Cell Signaling Technology; 1:1,000) and anti-glyceraldehyde 3-phosphate dehydrogenase (GAPDH; MAB374, Sigma Aldrich; 1:10,000). Membranes were then washed with TTBS and incubated with horseradish peroxidase-conjugated secondary antibodies (goat anti-rabbit IgG, #7074, Cell Signaling Technology; horse anti-mouse IgG, #7076; Cell Signaling Technology) diluted 1:2,000 in milk-TTBS at RT for 1 h. After washing with TTBS, the bands were visualized by enhanced chemiluminescence using SuperSignal West Pico Chemiluminescent Substrate (Thermo Scientific) and imaged with ChemiDoc MP Imaging System (Bio-Rad). Western Blots were quantified by measuring the optical density of the bands using ImageJ. GAPDH was used as a loading control and all detected bands were normalized to their corresponding GAPDH bands.

### RNA Isolation and qRT-PCR

For gene expression analysis, CMs on gelatin-coated 12-well plates were exposed to test compounds and ET-1 for 24 h. Cells were lysed in 500 µl of Trizol reagent (Invitrogen, Carlsbad, CA, United States), whereafter placed on ice before freezing at ‒80°C where stored (maximum one month) before RNA isolation. Total RNA was isolated using the Phase Lock Gel Heavy tubes (Quantabio, Beverly, MA, United States) according to the manufacturer’s instructions. RNA samples were treated with rDNase I according to manufacturer’s instructions (DNA-free Kit, AM1906, Invitrogen). RNA concentration and quality were measured by spectrophotometry with NanoDrop 1000 (Thermo Fisher Scientific, Waltham, MA, United States). cDNA was synthesized from 250–500 ng of total RNA in 10 µl reactions with Transcriptor First Strand cDNA Synthesis Kit (04897030001, Roche, Basel, Switzerland) according to the manufacturer’s protocol using random hexamer primers and MJ Mini Personal thermal cycler (Bio-Rad). The cDNA was diluted 1:10 in PCR grade H_2_O and stored at ‒20°C. Commercial TaqMan® Gene Expression Assays (Thermo Fisher Scientific) for *Nppa* (Rn00664637_g1), *Nppb* (Rn00580641_m1), β-actin coding gene *Actb* (Mm_00607939_s1), eukaryotic *18S rRNA* (4352930E), *NPPA* (Hs00383230_g1), *NPPB* (Hs01057466_g1) and *ACTB* (4333762T) were used with LightCycler® 480 Probes Master reagent (Roche) according to manufacturer’s instructions to analyze 4.5 µl of the cDNA dilution in 10 µl reactions on a white LightCycler® 480 Multiwell Plate 384 (04729749001, Roche) using a LightCycler® 480 Real-Time PCR System (Roche). Each reaction was run at least in duplicate and the average value of technical replicates was used in the analysis. The results were analyzed by the ΔΔCt method by normalizing first the quantification cycle (C_q_) values of the genes of interest to the average of the C_q_ values of reference genes *Actb/ACTB* and *18S rRNA* of the same sample and then normalizing the obtained ΔC_q_ values to the ΔC_q_ values of control sample. No-template controls were used to confirm absence of PCR contamination.

### Immunofluorescence Staining and High-Content Analysis

For high-content analysis (HCA), the cells on 96-well plates were exposed to the compounds and ET-1 or vehicle for 24 h or 48 h. Brefeldin A (Invitrogen) was added to the cells to be stained for pro-B-type natriuretic peptide (proBNP) 3 h before fixation to block exocytosis of proBNP-containing vesicles. The cells were washed twice with phosphate-buffered saline (PBS) and fixed with 4% paraformaldehyde at RT for 15 min and permeabilized with 0.1% Triton X-100 in PBS at RT for 10 min. Non-specific binding sites were blocked with 4% FBS in PBS for 45 min followed by addition of primary antibodies diluted in 4% FBS in PBS: sarcomeric α-actinin antibody (Sigma-Aldrich Cat# A7811, Lot# 128M4812V, RRID:AB_476766) at 1:200 or 1:600, cardiac troponin T antibody (Abcam Cat# ab45932, lot# GR3218549-1, RRID:AB_956386) at 1:800 and proBNP antibody (Abcam Cat# ab13115, Lot# GR3226067-3 RRID:AB_299694) at 1:200. After a 60-min incubation at RT, the cells were washed 3 × 5 min with PBS and incubated with Alexa Fluor®—conjugated secondary antibodies (Life Technologies, Eugene, Oregon) at 1:200, Alexa Fluor® -conjugated phalloidin (Life Technologies) at 1:50 and 4′,6-diamidino-2-phenylindole (DAPI; Sigma-Aldrich) at 1 µg/ml at RT for 45 min. The cells were then washed 3 × 5 min with PBS and stored at 4°C in PBS until imaged.

The CellInsight™ CX5 High Content Screening Platform (Thermo Scientific) was used to image and analyze the cells. Images were collected using a 10x objective (Olympus UPlanFL N 10x/0.3) with 16–25 sites per well to examine more than 100 cells in each well. The images were analyzed with the Cellomics software using two different protocols. The Cellomics Morphology Explorer BioApplication was used to measure α-actinin and F-actin staining intensity and to identify and measure the area and number of α-actinin and F-actin fibers in the CMs. Briefly, the CMs were identified based on α-actinin staining and DAPI staining was used to identify the nuclei of the CMs. Only CMs with one or two nuclei (mono- or binucleated cells) were included in the analysis. Average intensity of α-actinin and F-actin within each cell was measured. In addition, α-actinin and F-actin fibers were identified based on their intensity and morphology. The settings for fiber recognition were set manually for each experiment to allow optimal analysis regardless of minor variations in staining intensities. The fiber recognition and fiber area analyzed reflect cytoskeletal reorganization: increased fiber number and fiber area correspond to increased intensity and/or contrast compared to surrounding pixels, which results in increased recognition of fibers. The Cellomics Compartmental Analysis BioApplication was used to analyze the number of proBNP positive cells. First, the nuclei were identified based on DAPI staining and the average intensity of proBNP staining was then quantified from the perinuclear region defined as an 8-pixel ring around each nucleus. Cells were classified to proBNP positive and negative cells based proBNP intensity, and the reference level was set individually for each experiment. Each experiment included at least two technical replicates, i.e. two parallel wells of each treatment group. The average of technical replicates was used in the analysis.

### Statistical Analysis

The results are expressed as mean ± SD of at least three independent experiments. Each independent experiment was carried out with cells from individual cell isolations (NRVMs) or from individual differentiations (hiPSC-CMs). Each independent experiment, except Western blots, consisted of technical replicates, of which average was calculated for statistical analysis to represent n = 1. Single outliers of technical replicates were identified by Grubbs’ test (95% confidence) and removed from the data. The statistical analyses were performed using IBM SPSS Statistics 24 software. The one-way analysis of variance (ANOVA) and Tukey’s post-hoc tests were performed to analyze concentration-dependent effects of the compounds on cytotoxicity and cell viability and to analyze the effect of PKC agonists on expression of PKC isoforms. When unequal variances were detected by Levene’s test (*p* < 0.05), Welch’s ANOVA and Games-Howell post-hoc tests were used. For ERK phosphorylation, gene expression and HCA data, the statistical significance of two or three variables: ET-1, PKC activators and PKC or MEK1/2 inhibitors was evaluated by Univariate ANOVA. When unequal variances were detected by Levene’s test (*p* < 0.05), Kruskall-Wallis test was used. To locate the significantly differing groups, pairwise comparisons were performed by Student’s t-test for independent samples after Univariate ANOVA and by Mann-Whitney U test after Kruskall-Wallis test. To be able to perform the analysis, 1–2% artificial variance was added to the normalized data of the control group (DMSO). For the analysis of ERK phosphorylation, the data adjusted to GAPDH (but not normalized to DMSO) were used for statistical analysis. Values of *p* < 0.05 were considered statistically significant.

## Results

### Effects of PKC Modulators on Cardiomyocyte Viability

We first investigated the effects of PKC activators and inhibitors on the viability of CMs. None of the compounds exhibited significant toxicity in NRVMs or in hiPSC-CMs according to the LDH assay, cytotoxicity being always less than 10% (Supplementary Figure S1). The small increase in toxicity observed with HMI-1b11 treatment at the concentration of 30 µM (6.6 ± 6.0% in NRVMs and 5.4 ± 12% in hiPSC-CMs) may be due to poor solubility of the lipophilic compound at high concentration. In MTT assay, the cPKC inhibitor Gö6976 reduced metabolic activity in NRVMs (1 µM, 44 ± 7.1% of control, *p* < 0.001; 3 µM, 37 ± 6.7% of control, *p* < 0.001). The pan-PKC inhibitor Gö6983 had no significant effect on cell viability in the MTT assay. Bryostatin-1 increased metabolic activity at the concentration of 30 nM in hiPSC-CMs (151 ± 18%, *p* = 0.006) while in NRVMs the effect was less obvious. Of note, bryostatin-1 clearly induced beating of the cells (visual observation, not shown), which could in part explain the increased metabolic activity. In addition, there was a trend toward increased metabolic activity in both NRVMs and hiPSC-CMs with HMI-1b11 at the concentrations of 1–10 µM, but not at 30 µM. As both bryostatin-1 and HMI-1b11 activate PKC, the increase in metabolic activity might originate from increased PKC activity.

### Effects of PKC Modulators on ERK1/2 Phosphorylation

**FIGURE 1:**
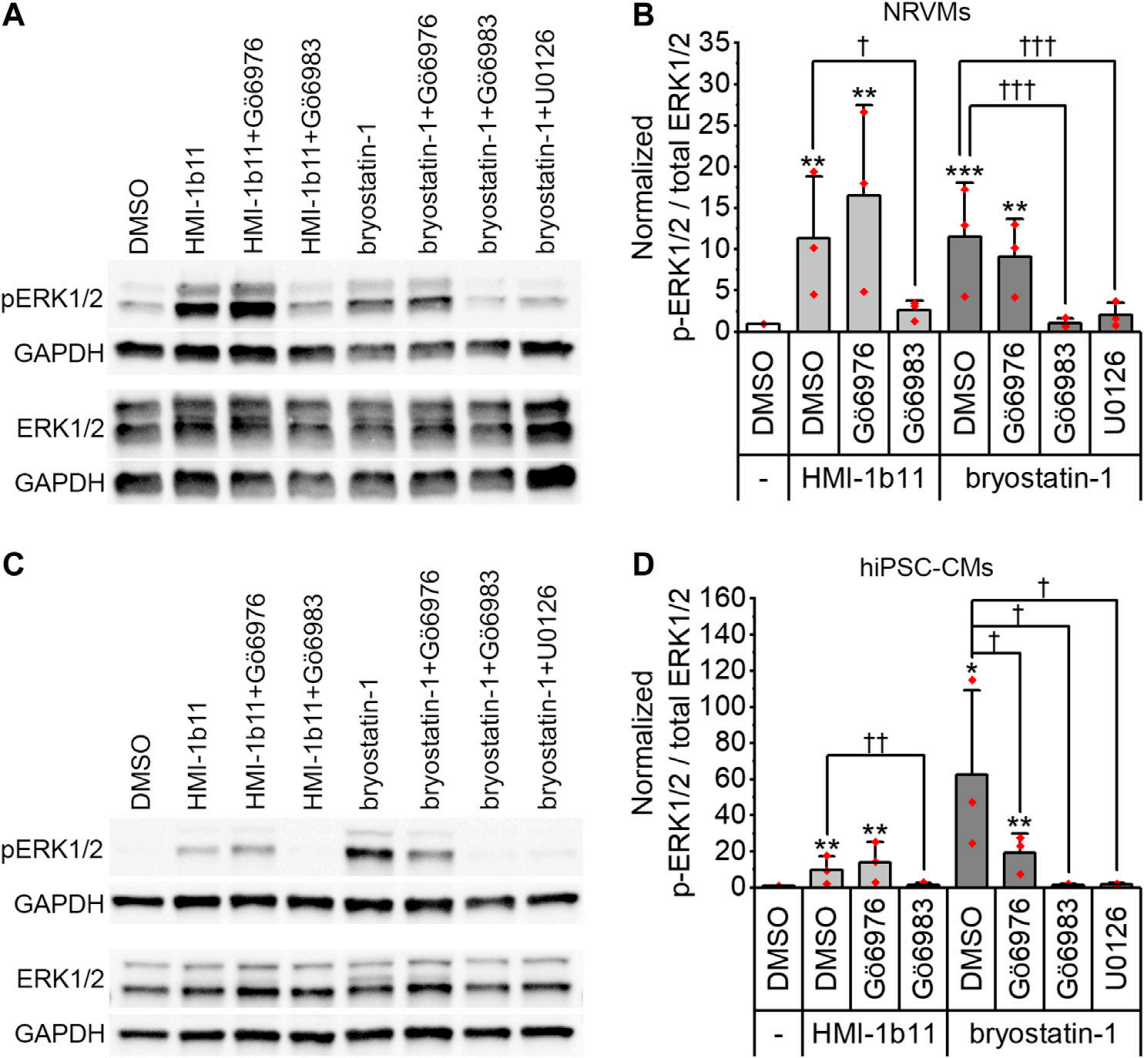
Effects of PKC modulators on ERK1/2 phosphorylation. Cells were exposed to cPKC inhibitor (1 µM Gö6976), pan-PKC inhibitor (1 µM Gö6983) or MEK1/2 inhibitor (10 µM U0126) for 15 min, after which PKC agonists HMI-1b11 (at 10 µM) or bryostatin-1 (at 10 nM) were added for 30 min. **(A)** Representative immunoblots for NRVMs; **(B)** quantification of Western blots expressed as mean + SD of three independent experiments (cardiomyocytes from individual cell isolations) for NRVMs; **(C)** representative immunoblots for hiPSC-CMs (day 18–20); **(D)** quantification of Western blots expressed as mean + SD of three independent experiments (cardiomyocytes from individual differentiations) for hiPSC-CMs. The expression of phosphorylated ERK1/2 (p-ERK1/2) and total ERK1/2 were normalized to GAPDH, after which the ratio was calculated and normalized to DMSO. ***p < 0.001, **p < 0.01, *p < 0.05 vs. DMSO; †††*p* < 0.001, ††*p* < 0.01, †*p* < 0.05 as indicated (Univariate ANOVA followed by student’s t-test for independent samples). The original full images of immunoblots are shown in Supplementary Figure S2.

To confirm that the PKC activators indeed activate PKC and induce PKC-mediated downstream signaling in hiPSC-CMs and NRVMs, phosphorylation of ERK1/2 was measured by Western blotting. Representative blots are shown for NRVMs and for hiPSC-CMs in Figures 1A,C, respectively. In both cell types, a 30-min treatment with PKC agonists (10 µM HMI-1b11 or 10 nM bryostatin-1) induced ERK1/2 phosphorylation (Figure 1). In hiPSC-CMs, the full agonist bryostatin-1 had a greater effect (62-fold vs. DMSO control, *p* = 0.043) than partial agonist HMI-1b11 (9.8-fold vs. DMSO control, *p* = 0.009), while in NRVMs the effects of two agonists were similar (11-fold vs. DMSO control, *p* < 0.001 for bryostatin-1, *p* = 0.008 for HMI-1b11). The effect of PKC agonists on ERK1/2 phosphorylation was inhibited by a 15-min pretreatment with the pan-PKC inhibitor Gö6983 or the MEK1/2 inhibitor U0126. In contrast, the cPKC inhibitor Gö6976 did not inhibit the response to PKC agonists in NRVMs, but attenuated the effect of bryostatin-1 on ERK1/2 phosphorylation in hiPSC-CMs: in the presence of Gö6976, bryostatin-induced ERK1/2 phosphorylation was 19-fold (*p* = 0.002) compared to DMSO. These results demonstrate that the PKC activators induce nPKC and MEK1/2 mediated ERK1/2 phosphorylation in both CM types.

### Effects of PKC Agonists on PKC Isoform Expression

**FIGURE 2:**
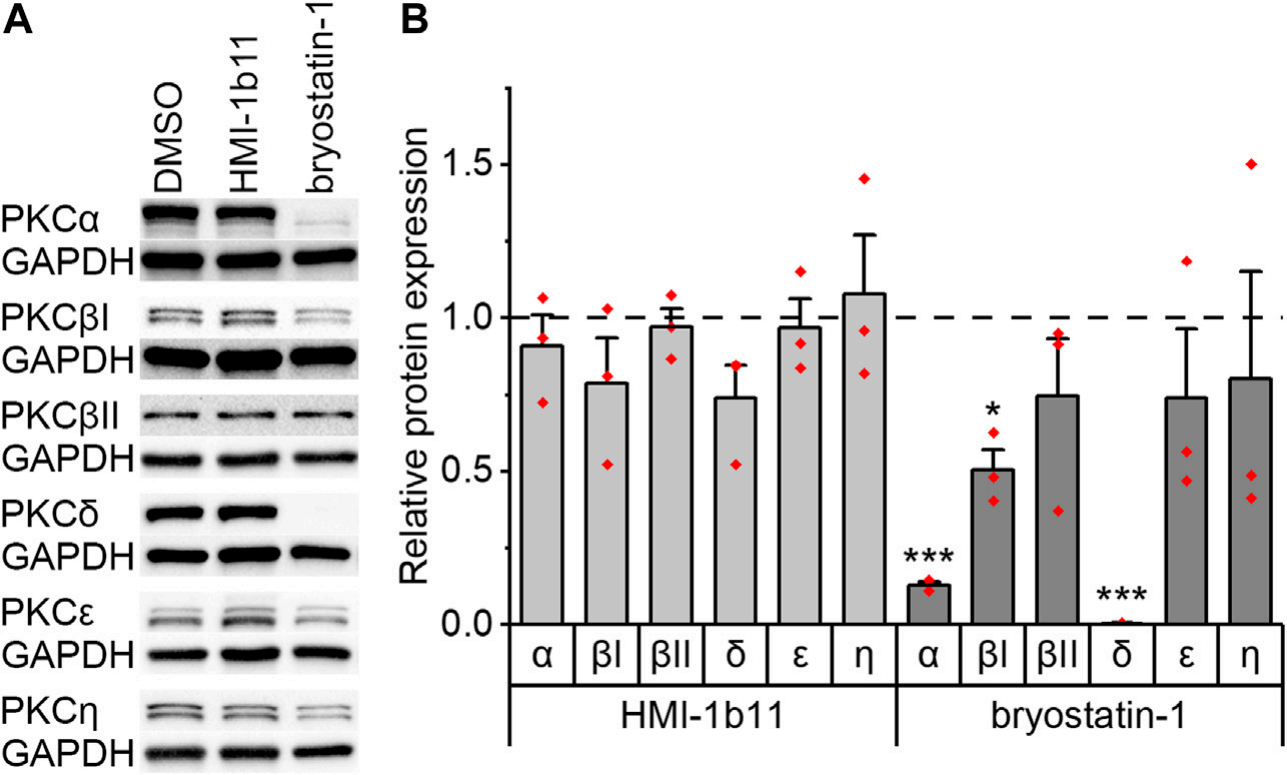
Effects of PKC agonists on the expression of PKC isoforms α, βI, βII, δ, ε and *η* in hiPSC-CMs. The cells were exposed to HMI-1b11 at 10 µM or bryostatin-1 at 10 nM for 48 h and proteins were detected by Western blotting. **(A)** Representative blots; **(B)** quantification of Western blots expressed as mean + SD of three independent experiments of cardiomyocytes from individual differentiations (day 57–182). Results are normalized to GAPDH and to DMSO control. ***p < 0.001, *p < 0.05 vs. DMSO control (Welch ANOVA followed by Games-Howell). The original full images of immunoblots are shown in Supplementary Figure S4.

The expression of PKC isoforms α, βI, βII, δ, ε, and η in hiPSC-CMs and in NRVMs was confirmed by Western blotting. Representative blots are shown for hiPSC-CMs in Figure 2A and for NRVMs in Supplementary Figure S3. As PKC activators have not previously been studied in hiPSC-CMs, the expression of PKC isoforms was studied after 48-h exposure to PKC agonists HMI-1b11 and bryostatin-1. The partial PKC agonist HMI-1b11 (at 10 µM) had no significant effect on the expression of any isoform, while the full agonist bryostatin-1, even at the low concentration of 10 nM, downregulated α, βI and δ isoforms (Figure 2). In particular, the expression of PKCδ was fully downregulated (0-fold vs. DMSO, *p* < 0.001), while the expression of PKCα and PKCβI was decreased 90% (*p* < 0.001) and 50% (*p* = 0.028), respectively. Bryostatin-1 had no effect on the expression levels of isoforms βII, ε and η.

### Effects of ET-1 on Hypertrophic Gene Expression

**FIGURE 3:**
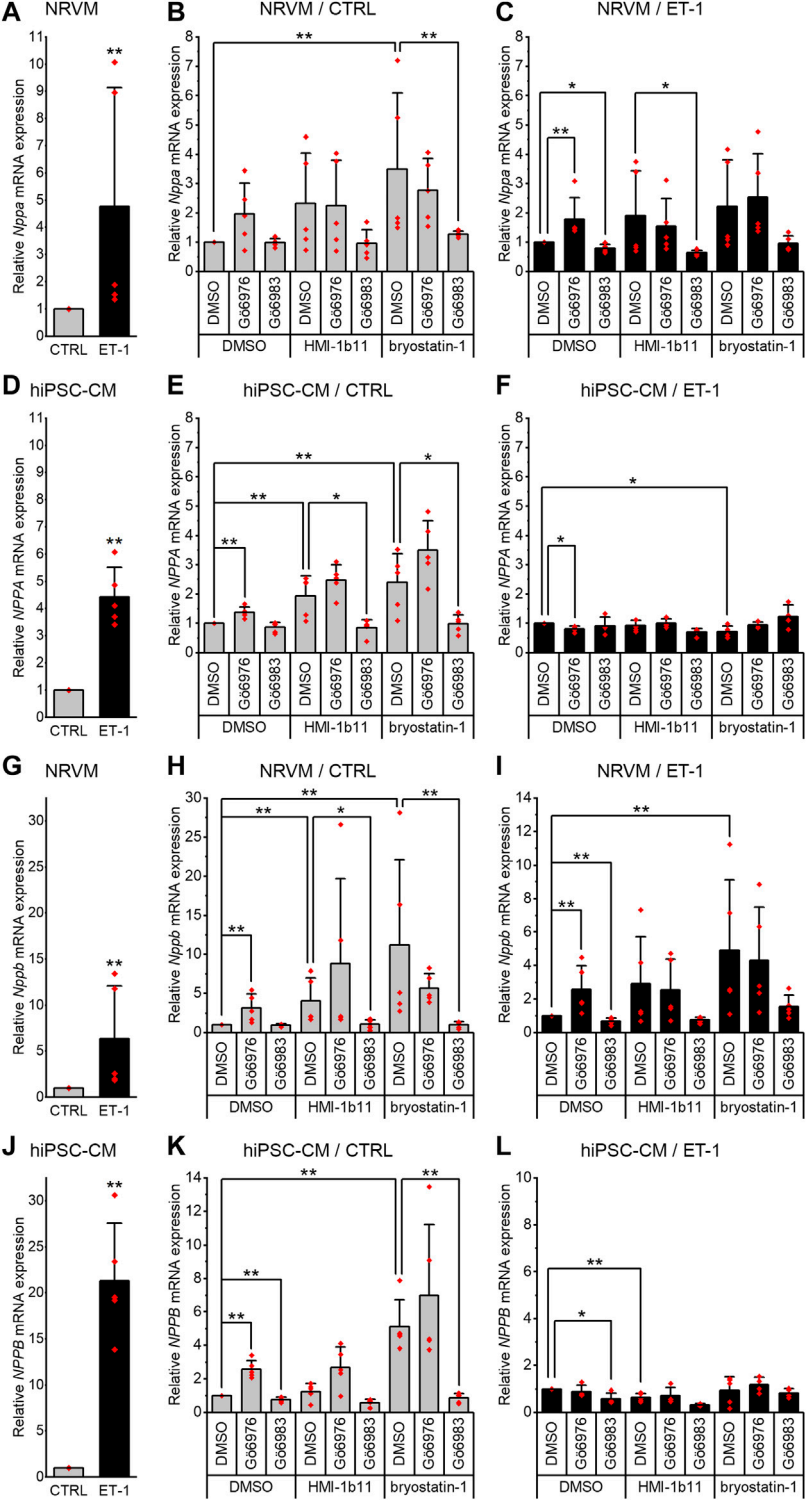
Effects of endothelin-1 (ET-1) and pharmacological PKC modulation on hypertrophic gene expression in cardiomyocytes. Relative *Nppa*/*NPPA* **(A–F)** and *Nppb*/*NPPB* **(G–L)** expression in NRVMs and in hiPSC-CMs was measured after 24-h exposure to PKC activators (10 µM HMI-1b11 and 10 nM bryostatin-1), cPKC inhibitor (1 µM Gö6976), pan-PKC inhibitor (1 µM Gö6983) with or without 100 nM ET-1. Results are expressed as mean + SD of independent experiments with cardiomyocytes from individual cell isolations or differentiations (n = 5 for NRVMs, n = 5 for hiPSC-CMs (day 31–52), except for inhibitors combined with ET-1 n = 4). ***p < 0.001, **p < 0.01, *p < 0.05 as indicated (Kruskall-Wallis followed by Mann-Whitney U).

To evaluate the hypertrophic responses of CMs, the expression of genes encoding natriuretic peptide A (*Nppa/NPPA*) and B (*Nppb/NPPB*) were analyzed. As expected, a 24-h ET-1 treatment increased hypertrophic gene expression in both cell types (Figure 3, left panels). ET-1 increased *Nppa/NPPA* expression similarly in both cell types, 4.8-fold (*p* = 0.008) in NRVMs and 4.4-fold (*p* = 0.008) in hiPSC-CMs (Figures 3A,D), while the increase in *Nppb/NPPB* expression was higher in hiPSC-CMs (21-fold, *p* = 0.008) than in NRVMs (6.3-fold, *p* = 0.008) (Figures 3G,J). These results show that ET-1-mediated hypertrophic signaling pathways are functional also in hiPSC-CMs.

### Effects of PKC Inhibition on Hypertrophic Gene Expression

Inhibition of all PKC isoforms by Gö6983 had no significant effect on the basal *Nppa or Nppb* gene expression in NRVMs (Figures 3B,H) but slightly reduced the ET-1-induced expression of *Nppa (*20%, *p* = 0.016) and *Nppb* (33%, *p* = 0.008) (Figures 3C,I). In hiPSC-CMs, the pan-PKC inhibitor Gö6983 had no significant effect on basal nor ET-1-induced *NPPA* expression (Figures 3E,F). However, Gö6983 decreased ET-1-induced *NPPB* expression in hiPSC-CMs by 42% (*p* = 0.016, Figure 3L) and also the basal *NPPB* gene expression by 23% (*p* = 0.008, Figure 3K). These results suggest that PKC is involved in ET-1-induced *Nppa* and *Nppb* signaling in NRVMs.

In contrast to inhibition of all PKC isoforms, cPKC inhibition with Gö6976 upregulated hypertrophic gene expression in both cell types (Figures 3H,K). However, for example, the upregulations of *Nppb/NPPB* caused by Gö6976 (3.1-fold, *p* = 0.008 in NRVMs and 2.6-fold, *p* = 0.008 in hiPSC-CMs), were considerably smaller than the changes induced by ET-1 (6.3-fold in NRVMs and 21-fold in hiPSC-CMs). In NRVMs, Gö6976 augmented ET-1-induced hypertrophic gene expression (*Nppa*, 1.8-fold increase, *p* = 0.008; *Nppb*, 2.6-fold increase, *p* = 0.008; Figures 3C,I), while in hiPSC-CMs cPKC inhibition with Gö6976 attenuated *NPPA* response to ET-1 by 20% (*p* = 0.016) and had no effect on *NPPB* response (Figures 3F,L). These results demonstrate that cPKC inhibition is pro-hypertrophic in NRVMs and hiPSCs in basal conditions. The effect is additive with ET-1-induced hypertrophic gene expression in NRVMs, but not in hiPSC-CMs.

### Effects of PKC Activation on Hypertrophic Gene Expression

In both cell types, the PKC activators increased hypertrophic gene expression (Figure 3, middle panels). The partial agonist HMI-1b11 increased *Nppb* expression by 4.1-fold (*p* = 0.008) in NRVMs and *NPPA* expression 1.9-fold (*p* = 0.008) in hiPSC-CMs. The more potent PKC activator, bryostatin-1, increased both *Nppa*/*NPPA* and *Nppb/NPPB* expression in NRVMs (3.5-fold, *p* = 0.008 for *Nppa* and 11-fold, *p* = 0.008 for *Nppb*) and in hiPSC-CMs (2.4-fold, *p* = 0.008 for *NPPA* and 5.1-fold, *p* = 0.008 for *NPPB*). Thus, in NRVMs, bryostatin-1 upregulated *Nppb* even more than ET-1, whereas in hiPSC-CMs the effect of ET-1 on hypertrophic gene expression was greater than that of any of the PKC-targeted compounds.

The augmentation of hypertrophic gene expression by PKC activators was observed also with concomitant treatment with ET-1 in NRVMs, but not in hiPSC-CMs (Figure 3, right panels). In fact, bryostatin-1 attenuated the *NPPA* expression by 27% (*p* = 0.016) and HMI-1b11 the *NPPB* expression by 36% (*p* = 0.008) in the presence of ET-1 in hiPSC-CMs (Figures 3F,L). These results indicate convergent but differential regulation of hypertrophic gene expression by PKC and ET-1 signaling in both CM types.

Inhibition of all PKC isoforms by Gö6983 reversed the effect of PKC activators on hypertrophic gene expression in both cell types (Figure 3). On the other hand, the inhibition of cPKC by Gö6976 tended to augment the effects of PKC activators in hiPSC-CMs, however, these changes were not statistically significant (Figures 3E,K). Overall, these results suggest that the PKC activators induce hypertrophic gene expression via nPKCs.

### Effects of PKC Modulators and ET-1 on proBNP Expression

**FIGURE 4:**
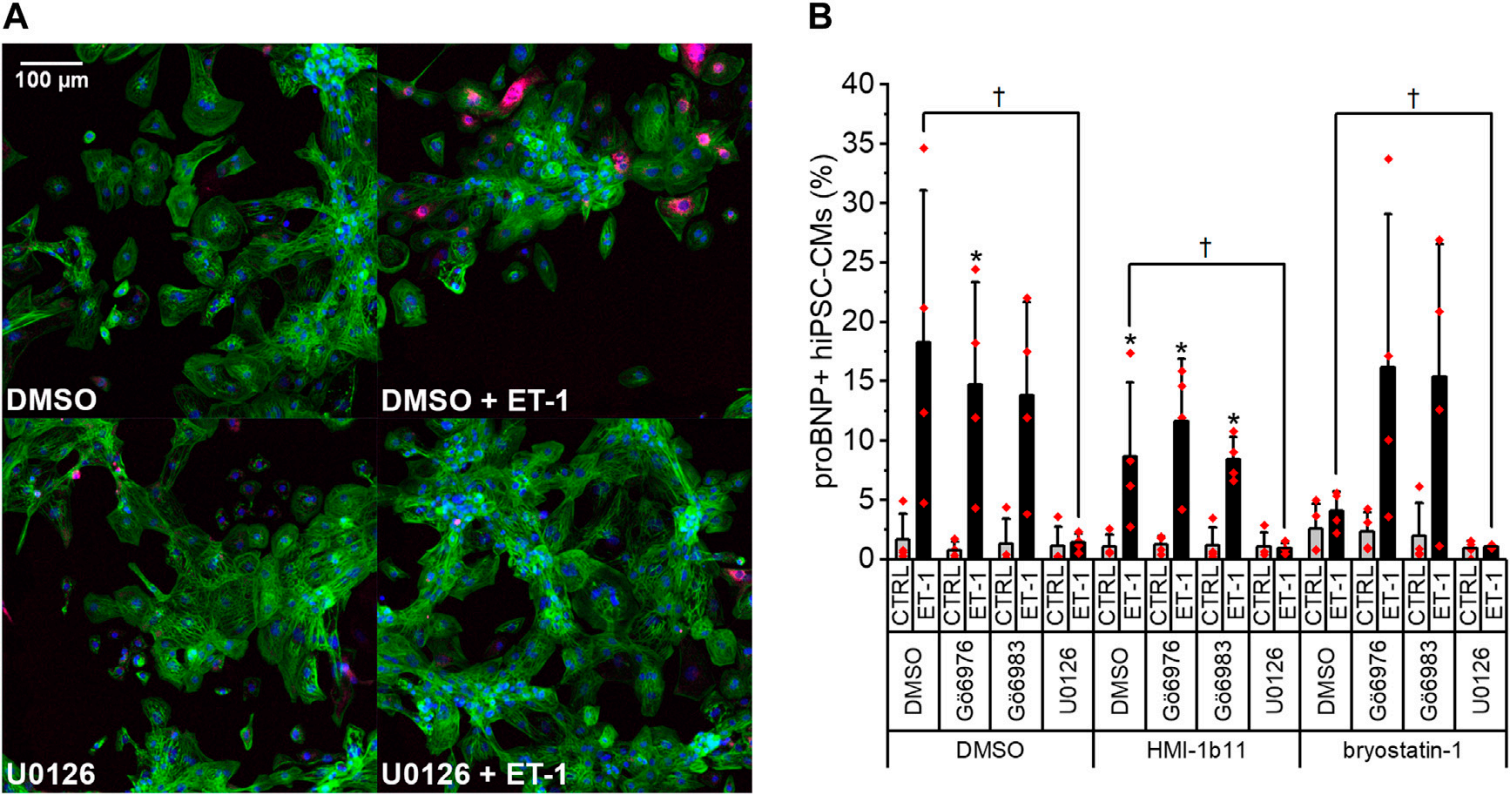
Effects of endothelin-1 (ET-1) and pharmacological PKC and MEK1/2 modulation on pro-B-type natriuretic peptide (proBNP) expression in human induced pluripotent stem cell-derived cardiomyocytes (hiPSC-CMs). **(A)** Representative images of DMSO, U0126 and ET-1 treated hiPSC-CMs stained for DAPI (blue), cardiac troponin T (green) and proBNP (magenta). **(B)** Percentage of proBNP positive cells is based on average intensity of proBNP staining in the perinuclear region after a 24-h exposure to PKC activators (10 µM HMI-1b11 and 10 nM bryostatin-1), cPKC inhibitor (1 µM Gö6976), pan-PKC inhibitor (1 µM Gö6983) and MEK1/2 inhibitor (10 µM U0126) with or without 100 nM ET-1. Results are expressed as mean + SD of four independent experiments with CMs from four individual differentiations (day 35–48). *p < 0.05 for ET-1 vs. respective CTRL; †*p* < 0.05 as indicated (Kruskall-Wallis followed by Mann-Whitney U).

To further study the effects of PKC modulators on hypertrophic response in hiPSC-CMs, expression of proBNP was studied by immunofluorescence staining and HCA. As the ERK1/2 pathway is a known downstream mediator of PKC and ET-1 signaling (Bogoyevitch et al., 1994), effects of the MEK1/2 inhibitor U0126 were examined in combination with PKC activators and ET-1. Representative immunofluorescence images in Figure 4A show a clear increase of proBNP positive cells in response to ET-1 and an inhibitory effect of the MEK1/2 inhibitor U0126. This was confirmed by quantification (Figure 4B): ProBNP was detected in the perinuclear region in 1.6 ± 2.2% of control hiPSC-CMs, whereas 18 ± 13% of ET-1-treated hiPSC-CMs were proBNP positive. None of the PKC-targeted compounds alone affected the percentage of proBNP positive cells significantly. However, PKC activator bryostatin-1 attenuated the ET-1-induced proBNP expression to 4.1 ± 1.6% (*p* = 0.114). Interestingly, this effect of bryostatin-1 was abolished with concomitant treatment with both pan-PKC and cPKC inhibitor. MEK1/2 inhibitor U0126 blocked ET-1-induced proBNP expression (to 0.6–1.4% proBNP positive cells) in both control and PKC agonist-treated cells. These results indicate that in hiPSC-CMs ET-1 signaling is mediated by MEK1/2, but not through PKC.

### Effects of PKC Modulators and ET-1 on Sarcomeric Proteins

**FIGURE 5:**
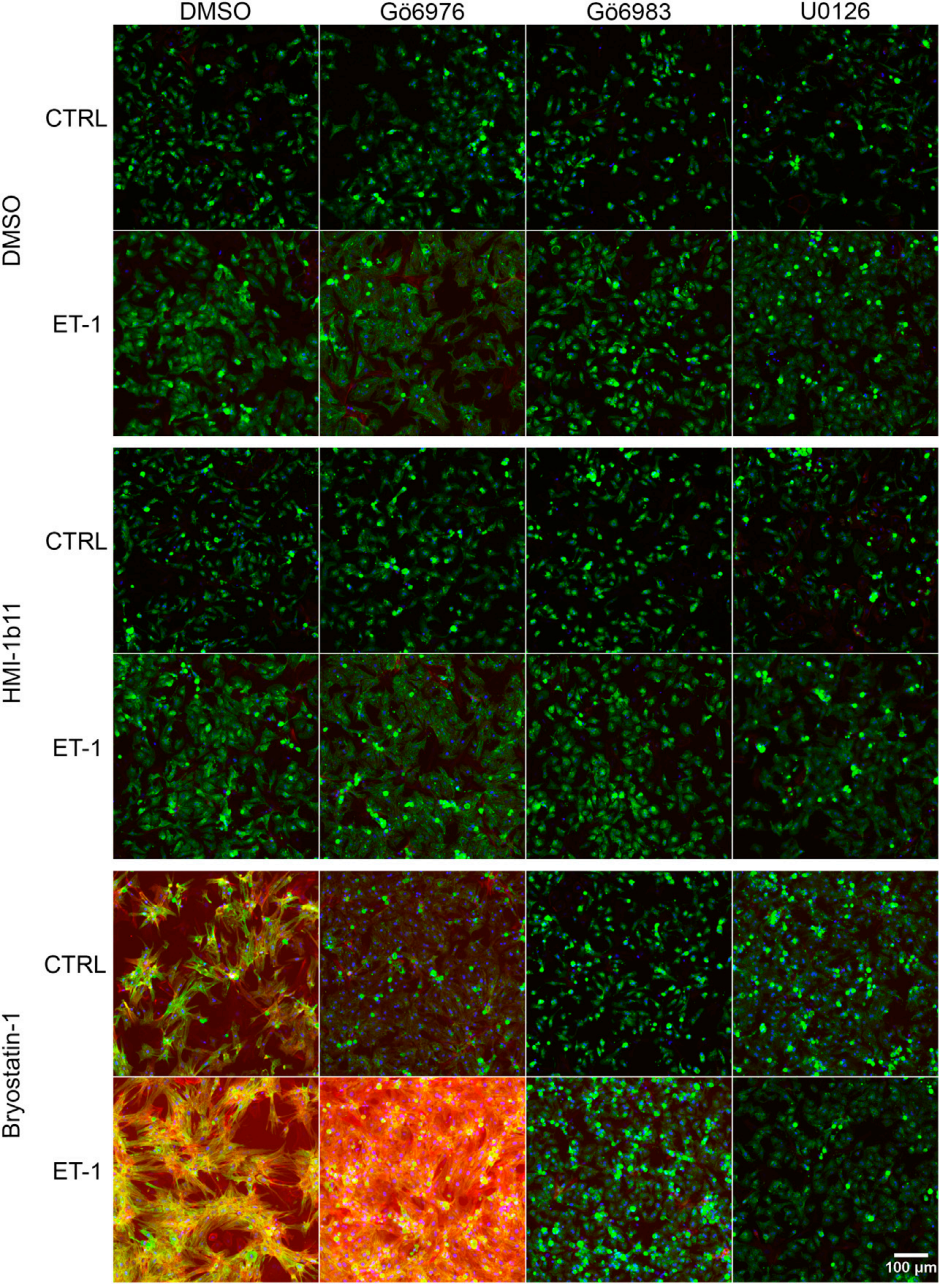
Effects of endothelin-1 (ET-1) and pharmacological PKC modulation on neonatal rat ventricular myocyte (NRVM) morphology. Representative images of immunofluorescence stained NRVMs treated with PKC activators (10 µM HMI-1b11 and 10 nM bryostatin-1), cPKC inhibitor (1 µM Gö6976), pan-PKC inhibitor (1 µM Gö6983) and MEK1/2 inhibitor (10 µM U0126) with or without 100 nM ET-1 for 48 h (blue = DAPI, green = α-actinin, red = F-actin). To better visualize morphology, see Supplementary Figure S5, where brightness and contrast have been adjusted individually for each image.

**FIGURE 6:**
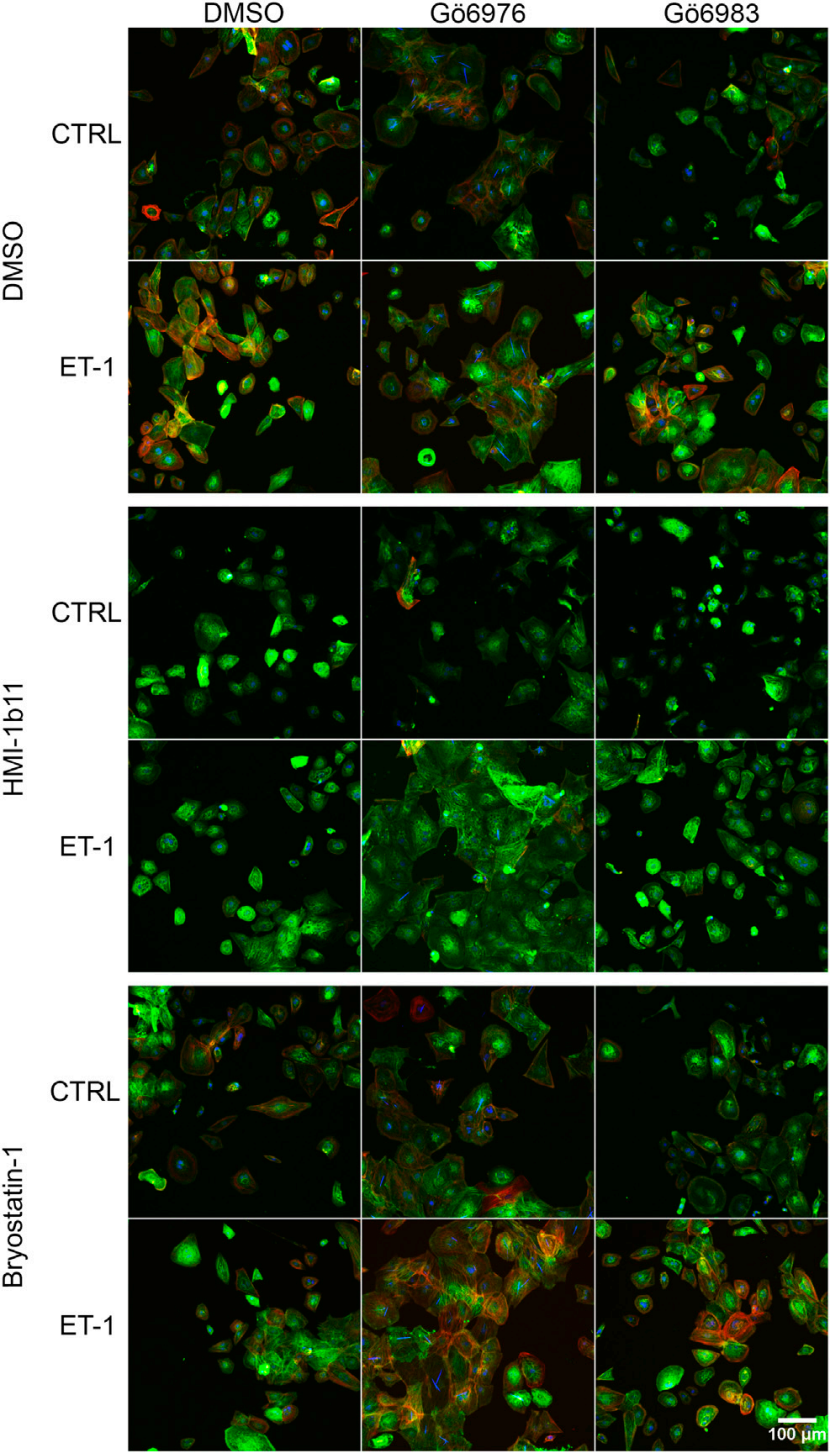
| Effects of endothelin-1 (ET-1) and pharmacological PKC modulation on human induced pluripotent stem cell-derived cardiomyocyte (hiPSC-CM) morphology. Representative images of immunofluorescence stained hiPSC-CMs (D33) treated with PKC activators (10 µM HMI-1b11 and 10 nM bryostatin-1), cPKC inhibitor (1 µM Gö6976) and pan-PKC inhibitor (1 µM Gö6983) with or without 100 nM ET-1 for 48 h (blue = DAPI, green = α-actinin, red = F-actin).

**FIGURE 7:**
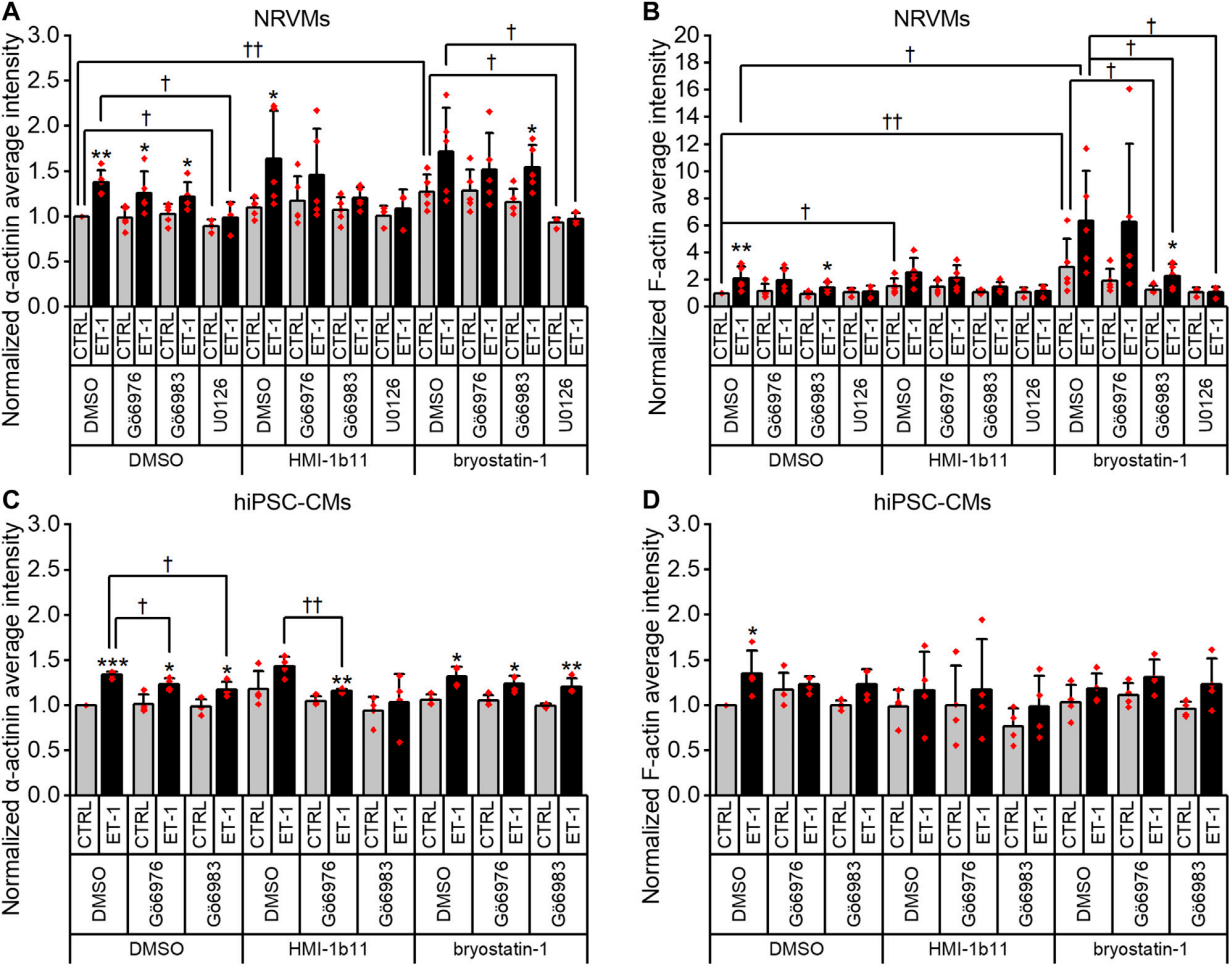
Effects of endothelin-1 (ET-1) and pharmacological PKC modulation on the average intensity of α-actinin and F-actin in cardiomyocytes. Average intensity of α-actinin and F-actin in NRVMs (**A,B**, respectively) and in hiPSC-CMs (**C,D**, respectively) was measured after 48 h exposure to PKC activators (10 µM HMI-1b11 and 10 nM bryostatin-1), cPKC inhibitor (1 µM Gö6976), pan-PKC inhibitor (1 µM Gö6983) and MEK1/2 inhibitor (10 µM U0126) with or without 100 nM ET-1. Results are normalized to DMSO control and expressed as mean + SD of independent experiments with CMs from individual cell isolations or differentiations (n = 4 for hiPSC-CMs (D32-D49), n = 5 for NRVMs except for U0126 n = 3). ***p < 0.001, **p < 0.01, *p < 0.05 for ET-1 vs. respective CTRL; ††*p* < 0.01, †*p* < 0.05 as indicated (Univariate ANOVA followed by student’s t-test for independent samples for hiPSC-CMs, Kruskall-Wallis followed by Mann-Whitney U for NRVMs).

To characterize the phenotypic hypertrophic changes induced by PKC modulators and ET-1, cardiomyocyte morphology was studied by analyzing α-actinin and F-actin fibers using HCA. Representative fluorescence microscopic images of NRVMs and hiPSC-CMs are shown in Figures 5, 6, respectively. The most striking effect was the dramatic increase in F-actin staining intensity in NRVMs treated with bryostatin-1, ET-1 and Gö6976 (Figures 5, 7B). ET-1 alone increased the average α-actinin and F-actin intensities in both cell types after a 48-h exposure (Figure 7). In NRVMs, ET-1 increased α-actinin average intensity by 1.4-fold (*p* = 0.008) and F-actin intensity 2.1-fold (*p* = 0.008). In hiPSC-CMs, the changes were slightly smaller; 1.3-fold change in α-actinin (*p* < 0.001) and 1.4-fold change in F-actin (*p* = 0.032). The hypertrophic effect of ET-1 was inhibited by U0126 (Figures 7A,B), indicating that the effect is MEK1/2-dependent in NRVMs (not studied in hiPSC-CMs). Inhibition of all PKC isoforms also attenuated the ET-1 response, in particular in hiPSC-CMs (Figures 7C,D). The PKC or MEK1/2 inhibitors alone had no effect on the average intensity of α-actinin or F-actin staining apart from a minor decrease (0.9-fold, *p* = 0.036) in α-actinin intensity in NRVMs after U0126 treatment. The PKC activators tended to increase α-actinin and F-actin intensity in NRVMs, bryostatin-1 having a greater effect than HMI-1b11. For example, bryostatin-1 increased the average α-actinin intensity by 1.3-fold (*p* = 0.008) and F-actin average intensity by 3.0-fold (*p* = 0.008).

**FIGURE 8:**
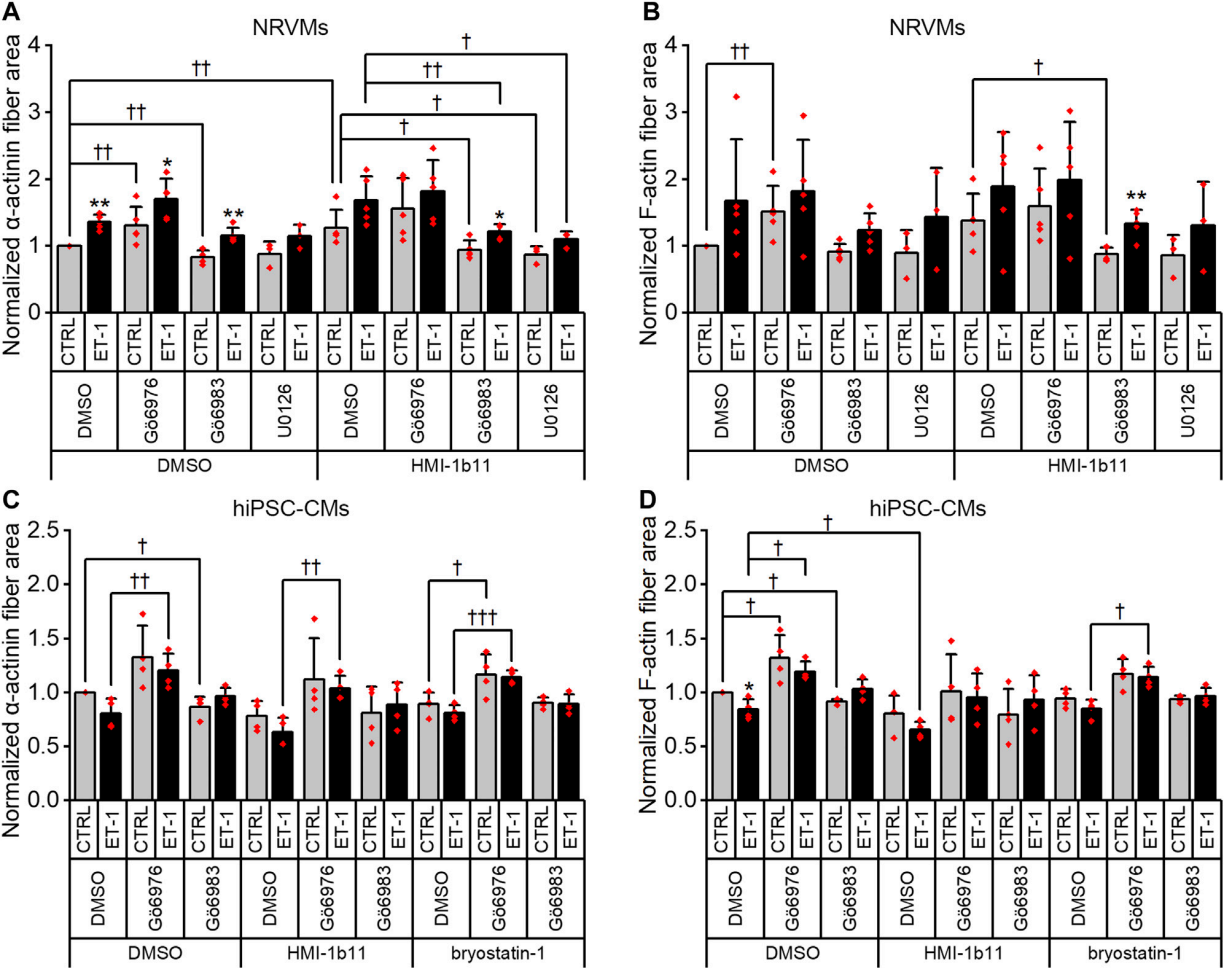
Effects of endothelin-1 (ET-1) and pharmacological PKC modulation on the area of α-actinin and F-actin fibers in cardiomyocytes. The area of α-actinin **(A,C)** and F-actin fibers **(B,D)** in NRVMs and in hiPSC-CMs was measured after 48 h exposure to PKC activators (10 µM HMI-1b11 and 10 nM bryostatin-1), cPKC inhibitor (1 µM Gö6976), pan-PKC inhibitor (1 µM Gö6983) and MEK1/2 inhibitor (10 µM U0126) with or without 100 nM ET-1. Results are normalized to DMSO control and expressed as mean + SD of independent experiments with CMs from individual cell isolations or differentiations (n = 4 for hiPSC-CMs (D32-D49), n = 5 for NRVMs, except for U0126 n = 3). **p < 0.01, *p < 0.05 for ET-1 vs. respective CTRL; †††*p* < 0.001, ††*p* < 0.01, †*p* < 0.05 as indicated (Kruskall-Wallis followed by Mann-Whitney U, except for α-actinin fiber area in hiPSC-CMs Univariate ANOVA followed by student’s t-test for independent samples).

Although α-actinin and F-actin average intensities were useful parameters to assess ET-1 response, they were not able to detect all effects seen for PKC modulators in hypertrophic gene expression. Therefore, we used HCA also to recognize α-actinin and F-actin fibers (Supplementary Figure S6) and to measure the area of recognized fibers (Figure 8). The ET-1 response was observed also in α-actinin and F-actin fiber area in NRVMs, but not in hiPSC-CMs: ET-1 increased the area of α-actinin and F-actin fibers in NRVMs (1.4-fold, *p* = 0.008 and 1.7-fold, *p* = 0.151, respectively), but decreased their area in hiPSC-CMs (0.8-fold, *p* = 0.063 and *p* = 0.029, respectively). The cPKC inhibitor Gö6976 increased α-actinin and F-actin fiber area both in NRVMs and in hiPSC-CMs, while the pan-PKC inhibitor Gö6983 slightly decreased the fiber area in both cell types. The MEK1/2 inhibitor U0126 had no significant effect on fiber area (studied only in NRVMs). Bryostatin-1 induced clumping of the NRVMs, which made the fiber analysis unfeasible. HMI-1b11, in turn, increased the α-actinin fiber area by 1.4-fold (*p* = 0.008) in NRVMs (Figure 8A). This effect was reversed by inhibition of all PKC isoforms or MEK1/2 inhibition. In hiPSC-CMs, PKC activators had no significant effect on fiber area. However, the effect of cPKC inhibitor Gö6976 was still observed in the presence of PKC activators, especially when combined with ET-1 (Figures 8C,D). The fiber recognition showed similar trends as the fiber area (Supplementary Figure S6). In conclusion, these results show that ET-1, PKC activation and cPKC inhibition cause increased recognition of α-actinin and F-actin fibers in NRVMs, while in hiPSC-CMs only the cPKC inhibition show similar effect. These results also confirm that while not being a high-resolution analysis of the sarcomeres, HCA is still able to detect general level cytoskeletal reorganization caused by hypertrophic stimuli.

### Effects of ET-1 and PKC Inhibitors on Cardiomyocyte Surface Area

**FIGURE 9:**
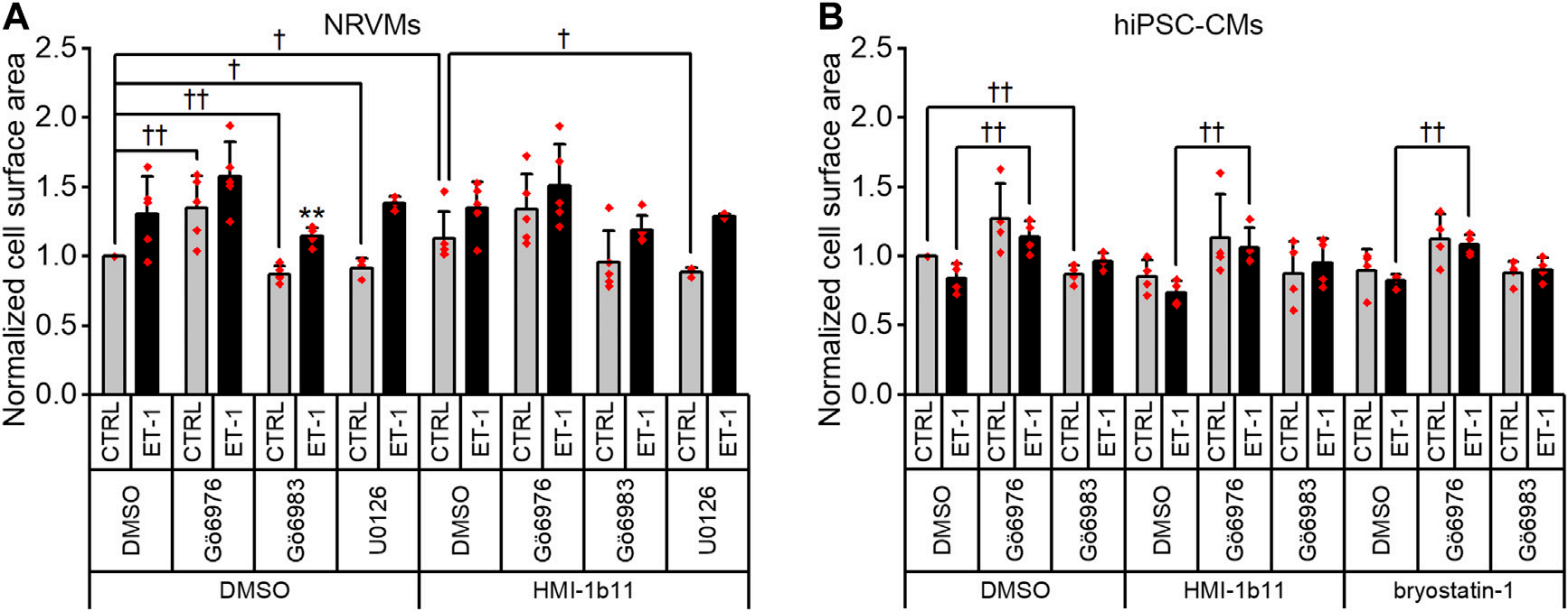
Effects of endothelin-1 (ET-1) and pharmacological PKC inhibition on cardiomyocyte cell surface area. Average cell surface area in NRVMs **(A)** and in hiPSC-CMs **(B)** was measured after a 48-h exposure to PKC activators (10 µM HMI-1b11 and 10 nM bryostatin-1), the cPKC inhibitor Gö6976 (1 µM), the pan-PKC inhibitor Gö6983 (1 µM) and MEK1/2 inhibitor (10 µM U0126) with or without 100 nM ET-1. Results are normalized to DMSO control and expressed as mean + SD of independent experiments with CMs from individual cell isolations or differentiations (n = 4 for hiPSC-CMs (D32-D49), n = 5 for NRVMs, except for U0126 n = 3). **p < 0.01 for ET-1 vs. respective CTRL; ††*p* < 0.01, †*p* < 0.05 as indicated (Univariate ANOVA followed by student’s t-test for independent samples for hiPSC-CMs and Kruskall-Wallis followed by Mann-Whitney U for NRVMs).

Finally, we measured cell surface area, a standard method to quantify CM hypertrophy. ET-1 tended to increase the cell surface area of NRVMs 1.3-fold (*p* = 0.151; Figures 5, 9A), but not that of hiPSC-CMs (Figures 6, 9B). Furthermore, the quantification of cell surface area was not feasible for NRVMs treated with bryostatin-1, which induced NRVMs to clump and stack up on top of each other (see Figure 5). The PKC activator HMI-1b11 increased the cell surface area of NRVMs by 1.1-fold (*p* = 0.032), and this was reversed by MEK1/2 inhibitor (*p* = 0.036). The hypertrophic effect induced by cPKC inhibition was observed in both cell types as measured by cell surface area, although in hiPSC-CMs statistically significantly only in the presence of ET-1.

## Discussion

PKC is associated with pathophysiology of many diseases, including left ventricular hypertrophy. Initially, different hypertrophic stimuli, including phorbol esters, α-adrenergic agonist and ET-1, were found to activate PKC (Shubeita et al., 1992; Bogoyevitch et al., 1994). In addition, overexpression of PKCα and PKCβ were shown to induce hypertrophic gene expression in CMs (Shubeita et al., 1992; Bowman et al., 1997; Braz et al., 2002). Later on, altered levels and activation of PKC, especially isoforms α, β, δ and ε, in hypertrophied and failed hearts supported the role of PKC in cardiac hypertrophy (Gu and Bishop, 1994; Bowling et al., 1999; Simonis et al., 2007). Furthermore, the role of different PKC isoforms in cardiomyocyte hypertrophy has been studied in rodents utilizing genetic manipulation and modulation of PKC isoform expression and translocation (Shubeita et al., 1992; Bowman et al., 1997; Wakasaki et al., 1997; Mochly-Rosen et al., 2000; Takeishi et al., 2000; Chen et al., 2001; Roman et al., 2001; Stebbins and Mochly-Rosen, 2001; Braz et al., 2002; Hahn et al., 2003; Braz et al., 2004; Gray et al., 2004; Klein et al., 2005; Liu et al., 2009; Song et al., 2015). For example, using isoform selective translocation inhibitors, Stebbins and Mochly-Rosen demonstrated that inhibition of PKCβI or PKCβII translocation inhibits phorbol 12-myristate 13-acetate (PMA)-induced hypertrophy in neonatal rat CMs *in vitro*, suggesting pro-hypertrophic roles for PKCβI and PKCβII (Stebbins and Mochly-Rosen, 2001). Mochly-Rosen et al. also showed that inhibition of PKCε translocation had a dose-dependent inhibitory effect on normal postnatal myocardial development in mice, causing lethal cardiac dilatation and CM hypertrophy (Mochly-Rosen et al., 2000). In addition, a PKCε translocation activator was shown to increase myocardial growth and ventricular remodeling with preserved cardiac function and without increase in CM size, suggesting that PKCε contributes to physiological hypertrophy and developmental hyperplasia. In contrast, Song et al. (2015) showed that mice with double cardiac knockout of PKCδ and PKCε had greater hemodynamic overload-induced cardiac hypertrophy and dysfunction than single PKCδ or PKCε knockout mice or wild type mice, suggesting that these nPKCs actually limit CM growth with functional redundancy. Overall, these studies have suggested both classical isoforms βI and βII and novel isoforms δ and ε to regulate hypertrophic responses.

The conflicting results gained with different methods may be explained by cross-regulation and redundancy of PKC isoforms. For example, when one PKC isoform is deleted or inhibited, another isoform may replace it, as in the case when knockout of PKCε increased the expression of PKCδ (Klein et al., 2005) or when silencing of PKCα led to the activation of PKCδ (Naskar et al., 2014). Furthermore, long-term PKC activation with ultrapotent agonists, such as phorbol esters, often leads to down-regulation of PKC protein levels, and thus does not correspond to the effects of physiological PKC activation (Newton and Brognard, 2017). Hence, elucidating the role of PKC and the effects of PKC modulators on CM hypertrophy will not only facilitate the development of novel therapies to cardiac diseases, but will also give insight into the cardiac effects of PKC modulators developed for other applications, such as for the treatment of cancer. In this study, the role of PKC in ET-1-induced cardiomyocyte hypertrophy was examined using pharmacological tools, including two PKC activators acting on classical and novel PKC isoforms, a cPKC inhibitor and a pan-PKC inhibitor.

Because PKC isoforms may have divergent roles in different species (Weeks and McMullen, 2018) and hiPSC-CMs represent a unique possibility to study human cardiomyocytes, we used both hiPSC-CMs and NRVMs in the present study. According to the present results, PKC modulators have similar effects in both cell types, suggesting that PKCs have comparable roles in the hypertrophy of both human and rat CMs. The current changes in hypertrophic gene expression and phenotypic parameters quantified with HCA suggest that pharmacological PKC activators indeed induce CM hypertrophy. Moreover, since inhibition of all PKC isoforms reversed the effect, the effect was PKC-dependent and not mediated by another C1 domain-containing protein (see Das and Rahman, 2014). Furthermore, the degree of agonism was also reflected in the responses, as the very potent full agonist bryostatin-1 had greater effects than the partial agonist HMI-1b11. In contrast to inhibition of all PKC isoforms, inhibition of cPKCs did not inhibit PKC activator-induced hypertrophy, but appeared to promote hypertrophy and aggravate PKC activator-induced hypertrophy. It should be noted that bryostatin-1 induced downregulation of PKC isoforms α, βI and δ under the experimental conditions used for phenotypic analyses in this study, while HMI-1b11 had no effect on PKC protein levels. Since the responses to bryostatin-1 and HMI-1b11 were similar, it can be speculated that these isoforms may not be responsible for the pro-hypertrophic effect. Our results thus suggest that the nPKCs, possibly PKCε and/or PKCη, are responsible for mediating the PKC agonist-induced hypertrophic response. Furthermore, cPKCs might have an anti-hypertrophic role in CMs, so that when cPKCs are inhibited, the balance is moved toward activation of nPKCs, which then could mediate the hypertrophic response. This is in conflict with a previous report showing that knockdown of PKCα inhibited ET-1-induced hypertrophy (Kerkelä et al., 2002). The current data suggesting a pro-hypertrophic role of nPKCs also conflicts with findings reported by Song et al. (2015). However, the knockout of PKC used by Song et al. (2015) and pharmacological inhibition of PKC activity used in the present study are not necessary expected to yield similar biological responses, as some of PKC-mediated processes are independent of kinase activity and are instead mediated by protein-protein interactions (Cameron and Parker, 2010). Furthermore, compensatory mechanisms activated by catalytic modulation of PKC activity and knockdown of PKC protein expression are expected to be different. Furthermore, it is noteworthy that the cPKC inhibitor Gö6976 also inhibits protein kinase D (PKD), another known regulator of CM growth. PKD activity is, however, considered pro-hypertrophic and PKD knockdown inhibits CM hypertrophy and cardiac remodeling (Fielitz et al., 2008; Sin and Baillie, 2012), and therefore, the present results are not likely PKD-mediated. Although PKC activation was recently shown to have antifibrotic effect in cardiac fibroblasts suggesting potential strategy to inhibit myocardial fibrosis (Karhu et al., 2020a), the pro-hypertrophic role of PKC agonists should be considered.

Although both NRVMs and hiPSC-CMs responded similarly to PKC modulators, these two CM types differed in their ET-1 signaling. In ET-1-stimulated hiPSC-CMs, the effects of PKC modulators were abolished, while they were still observed in ET-1-stimulated NRVMs. Thus, the effects of ET-1 might be mediated through different signaling pathways in NRVMs and hiPSC-CMs. In view of the present results, in hiPSC-CMs, ET-1-signaling passes through other pathways than PKC. However, as the MEK1/2 inhibitor blocked the ET-1-induced increase in proBNP expression in hiPSC-CMs, this PKC-independent signaling could still be mediated through MEK1/2, potentially by direct activation of ERK-MEK1/2 by Gα_q_. In contrast, in NRVMs, the response to ET-1 does not seem to be mediated neither solely through PKC nor MEK1/2 signaling, since inhibition of all PKC isoforms or MEK1/2 could not fully prevent the hypertrophic gene expression or the phenotypic response to ET-1. These results agree with the observation by Strait et al. (2001) that neither PKCε nor ERK1/2 are necessary for ET-1-induced hypertrophy. In addition, Ueyama et al. (2000) showed that dominant negative MEK1 attenuated the response to different hypertrophic stimuli, including ET-1, phenylephrine, leukemia inhibitory factor and isoproterenol, but could not fully inhibit hypertrophic response. Alternatively, ET-1 may act by activating calcium dependent pathways (calcineurin or calcium/calmodulin-dependent protein kinase II) or other not fully characterized hypertrophy-associated pathways, e.g. phosphoinositide 3-kinase p110γ pathway or other mitogen-activated protein kinase cascades.

NRVMs and hiPSC-CMs have very different phenotypes. Although NRVMs do not represent adult CMs, neither do hiPSC-CMs, which have more structural and functional similarities to embryonic CMs (Robertson et al., 2013). For instance, their sarcomeric structure, metabolism and calcium handling differ from adult CMs. HiPSC-CMs, however, beat spontaneously and synchronously, express several cardiac specific genes and proteins and share many electrophysiological characteristics to adult CMs. Therefore, while the cell surface area can be used as a phenotypic readout of hypertrophy in rodent CMs, the flat and rounded phenotype of hiPSC-CMs makes it less feasible to detect cell growth by measuring cell surface area, as also shown here. Therefore, we analyzed hiPSC-CM hypertrophy using HCA to quantify the expression of proBNP. This method has previously been developed to screen for ET-1-induced hypertrophic phenotype of hiPSC-CMs (Carlson et al., 2013) and has also been used to investigate acute and subacute effects of doxorubicin in hiPSC-CMs (Karhu et al., 2020b). In the present study, the increased abundance of proBNP-containing vesicles in response to ET-1 was successfully quantified, but the effects of PKC modulators that were seen in *NPPB* mRNA level were not detected with proBNP staining. Therefore, we quantified α-actinin and F-actin intensity and fibers. These readouts correlated well with hypertrophic gene expression in NRVMs, but not in hiPSC-CMs. The average intensity of α-actinin or F-actin was increased by ET-1 in hiPSC-CMs, but again, as in the proBNP assay, effects of PKC modulators were not detected. The only responses to PKC modulators in HCA were seen in the cell surface area and fiber analysis, in which cell surface area and recognition and area of α-actinin and F-actin fibers were increased by cPKC inhibition. However, no clear morphological responses to ET-1 or PKC activators were detected in hiPSC-CMs. Since the morphological changes were not apparent by visual observation, it can be speculated that while the hiPSC-CMs seem to exhibit appropriate gene expression responses to ET-1 stimulation and PKC modulation, their structural immaturity may prevent the development of clear morphological hypertrophy and upregulation of sarcomeric proteins. It is possible that more sensitive analysis using high-resolution confocal microscopy of sarcomeric structures or volumetric analysis of hiPSC-CMs could be used to detect more subtle changes in hiPSC-CM morphology.

## Conclusion

**FIGURE 10:**
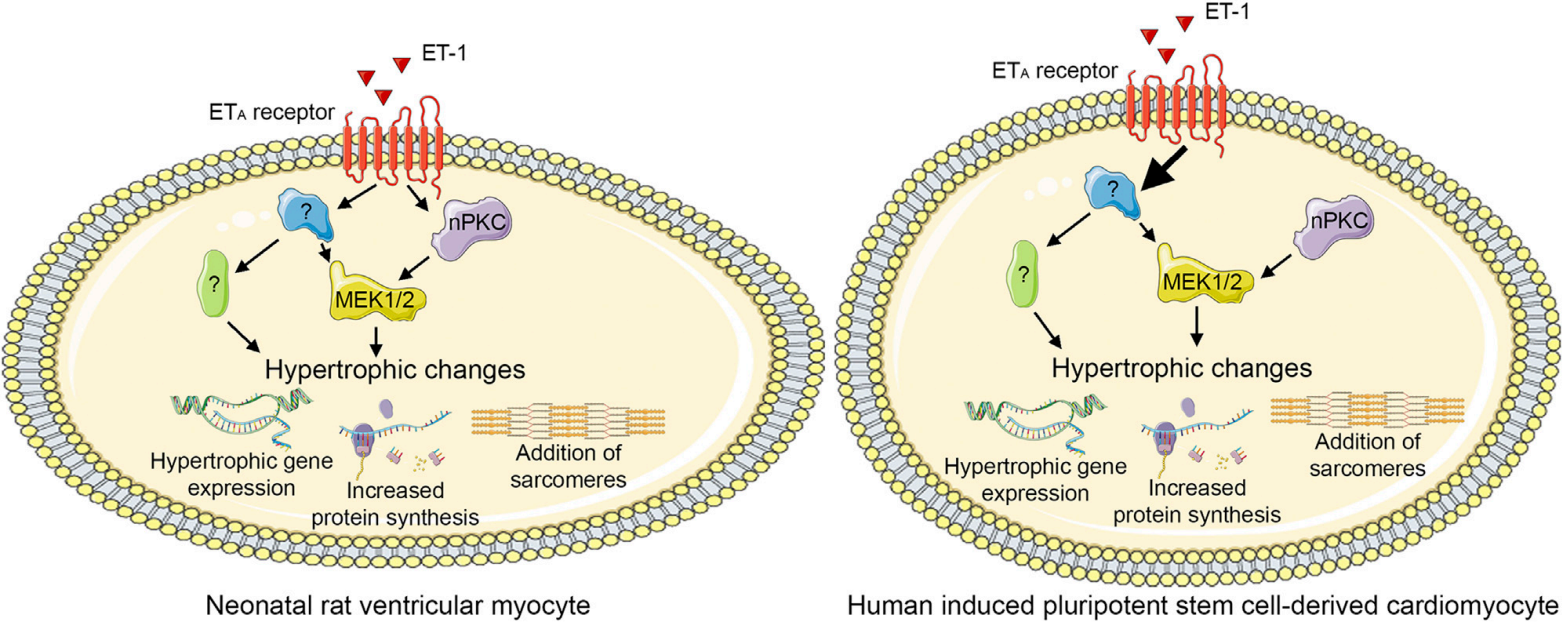
Proposed PKC-mediated hypertrophic signaling. Endothelin-1 (ET-1) induces hypertrophy both via novel protein kinase C (nPKC) isoforms and mitogen-activated kinase kinase 1/2 (MEK1/2) and via other, PKC-and MEK1/2-independent, mechanism(s) in neonatal rat ventricular cardiomyocytes. In human induced pluripotent stem cell-derived cardiomyocytes, ET-1 induces hypertrophy independent of PKC, although also nPKC activation induces hypertrophy. This figure was created using images from Servier Medical Art (http://smart.servier.com). Servier Medical Art by Servier is licensed under a Creative Commons Attribution 3.0 Unported License (https://creativecommons.org/licenses/by/3.0/).

To our best knowledge, the role of PKC isoforms in cardiomyocyte hypertrophy has not been investigated previously using hiPSC-CMs. Our present results show that PKCs have similar roles in hypertrophy of hiPSC-CMs and NRVMs: CM hypertrophy can be induced by either activation of PKC or inhibition of cPKCs, indicating a pro-hypertrophic role for nPKCs. Furthermore, this study provides further evidence on PKC-independent mechanisms of ET-1-induced CM hypertrophy both in NRVMs and in hiPSC-CMs. Moreover, different responses to PKC modulators in the presence of ET-1 suggest that dissimilarity in molecular pathways exist in rat and human CMs (Figure 10). Importantly, due to the morphological differences between NRVMs and hiPSC-CMs, similar phenotypic analysis readouts could not be reliably applied to compare hypertrophic responses between the cell types, and thus more comprehensive HCA methods are needed to assess morphological changes occurring in hiPSC-CM hypertrophy. Our results highlight the differences between the two CM types, which should be taken into consideration, when applying rodent studies to human. Furthermore, the present results strengthen the evidence on pro-hypertrophic effects of PKC activators, which are currently being developed for various indications such as cancer and Alzheimer’s disease thus indicating that potent PKC activators exhibit a significant risk of cardiac adverse effects.

## Data Availability Statement

The raw data supporting the conclusions of this article will be made available by the authors, without undue reservation.

## Author Contributions

VT and HR conceived the experiments and supervised the project. LP, JE, and RS carried out the experiments and analyzed the data. LP wrote the manuscript with support from VT and HR. All authors discussed the results and contributed to the final manuscript.

## Funding

This work was supported by the Finnish Foundation for Cardiovascular Research; the Academy of Finland (grant numbers 2666621 and 321564); Business Finland (3iRegeneration project, grant number 40395/13), the Sigrid Jusélius Foundation; and the Doctoral Programme in Drug Research.

## Conflict of Interest

The authors declare that the research was conducted in the absence of any commercial or financial relationships that could be construed as a potential conflict of interest.

## **Abbreviations:**

aPKC, atypical protein kinase C; CM, cardiomyocyte; cPKC, classical protein kinase C; CM, DAG, diacylglycerol; ERK1/2, extracellular signal-regulated kinase 1 and 2; ET-1, endothelin-1; GAPDH, glyceraldehyde 3-phosphate dehydrogenase; HCA, high-content analysis; hiPSC, human induced pluripotent stem cell; hiPSC-CM, human induced pluripotent stem cell-derived cardiomyocyte; LDH, lactate dehydrogenase; MEK1/2, mitogen-activated kinase kinase 1/2; MTT, 3-(4,5-dimethyl-2-thiazolyl)-2,5-diphenyltetrazolium bromide; nPKC, novel protein kinase C; Nppa/NPPA, natriuretic peptide A (rodent/human); Nppb/NPPB, natriuretic peptide B (rodent/human); NRVM, neonatal rat ventricular myocyte; PKC, protein kinase C; PKD, protein kinase D; proBNP, pro-B-type natriuretic peptide; PS, phosphatidylserine; qRT-PCR, quantitative reverse transcription PCR; RACK, receptor for activated C kinase.
