# Supplementary material for: Pharmacological Protein Kinase C Modulators Reveal a Pro-hypertrophic Role for Novel Protein Kinase C Isoforms in Human Induced Pluripotent Stem Cell-Derived Cardiomyocytes
Source: Front Pharmacol. 2021 Jan 20;11:553852. doi: 10.3389/fphar.2020.553852 (PMC7874215; doi:10.3389/fphar.2020.553852)
Supplement: Supplementary file 1 [file datasheet1.pdf]

Supplementary Material

1 Figure legends for Supplementary Figures

Supplementary Figure S1. Effects of PKC activators and inhibitors on cardiomyocyte cytotoxicity and viability. Lactate dehydrogenase (LDH) assay was used to measure cytotoxicity (**A** and **C**) and 3-(4,5-dimethyl-2-thiazolyl)-2,5-diphenyltetrazolium bromide (MTT) assay was used to evaluate the viability (**B** and **D**) of neonatal rat ventricular myocytes (NRVMs) and human induced pluripotent stem cell-derived cardiomyocytes (hiPSC-CMs) after 24 h exposure to the compounds. Results are expressed as mean±SD of independent experiments using CMs from individual cell isolations or differentiations (n=3 for hiPSC-CMs (day 19-30) and for PKC inhibitors in NRVMs, n=4 for PKC activators in NRVMs, n=6 for DMSO in NRVMs). ***p<0.001, **p<0.01, *p<0.05 vs. 0.3% DMSO control (shown as 0 nM or 0 µM; One-way ANOVA followed by Tukey).

Supplementary Figure S2. Original digital images of Western blots of phosphorylated ERK1/2 (pERK) and total ERK1/2 used for analysis shown in Figure 1. The membranes were first blotted for pERK or ERK, after which the same membranes were blotted for loading control GAPDH, which is shown below its corresponding pERK or ERK blot. **A**-**D** for NRVMs and **E**-**H** for hiPSC-CMs (day 19).

Supplementary Figure S3. Digital images of Western blots for analysis of PKC isoform expression in NRVMs. NRVMs were lysed after three days of culturing. Samples from three different cell isolations (1-3) were collected. GAPDH was used as a loading control and corresponding GAPDH bands are shown for each PKC isoform blot.

Supplementary Figure S4. Original digital images of Western blots for analysis of PKC isoform expression in hiPSC-CMs (day 57-182). Control samples from three independent experiment (CMs from individual cell differentiations) are numbered 1, 4 and 7. Corresponding HMI-1b11-treated samples are numbered 2, 5 and 8, while bryostatin-1-treated samples are numbered 3, 6 and 9. GAPDH was used as a loading control and corresponding GAPDH bands are shown below each PKC isoform blot.

Supplementary Figure S5. Effects of endothelin-1 (ET-1) and pharmacological PKC modulation on neonatal rat ventricular myocyte (NRVM) morphology. Representative images of immunofluorescence stained NRVMs treated with PKC activators (10 µM HMI-1b11 and 10 nM bryostatin-1), cPKC inhibitor (1 µM Gö6976), pan-PKC inhibitor (1 µM Gö6983) and MEK1/2 inhibitor (10 µM U0126) with or without 100 nM ET-1 for 48 h (blue=DAPI, green=α-actinin, red=F-actin). The intensities are not comparable across images, as brightness and contrast have been adjusted individually for each image.

Supplementary Figure S6. Effects of endothelin-1 (ET-1) and pharmacological PKC modulation on α-actinin and F-actin fiber recognition in cardiomyocytes. High content analysis was used to quantify the number of recognized α-actinin (**A** and **C**) and F-actin fibers (**B** and **D**) in neonatal rat ventricular

myocytes (NRVMs) and in human induced pluripotent stem cell-derived cardiomyocytes (hiPSC-CMs) after a 48-h exposure to PKC activators (10 µM HMI-1b11 and 10 nM bryostatin-1), cPKC inhibitor (1 µM Gö6976), pan-PKC inhibitor (1 µM Gö6983) and MEK1/2 inhibitor (10 µM U0126) with or without 100 nM ET-1. The data does not represent the actual number of fibers but the number of recognized fibers by high content analysis and increased values thus reflect cytoskeletal reorganization upon hypertrophic stimuli. Results are normalized to control and expressed as mean±SD of independent experiments with CMs from individual cell isolations or differentiations (n=4 for hiPSC-CMs (day 30-49), n=5 for NRVM, except for U0126 n=3).
