# Supplementary figures and images for: Pharmacological Protein Kinase C Modulators Reveal a Pro-hypertrophic Role for Novel Protein Kinase C Isoforms in Human Induced Pluripotent Stem Cell-Derived Cardiomyocytes
Source: Front Pharmacol. 2021 Jan 20;11:553852. doi: 10.3389/fphar.2020.553852 (PMC7874215; doi:10.3389/fphar.2020.553852)

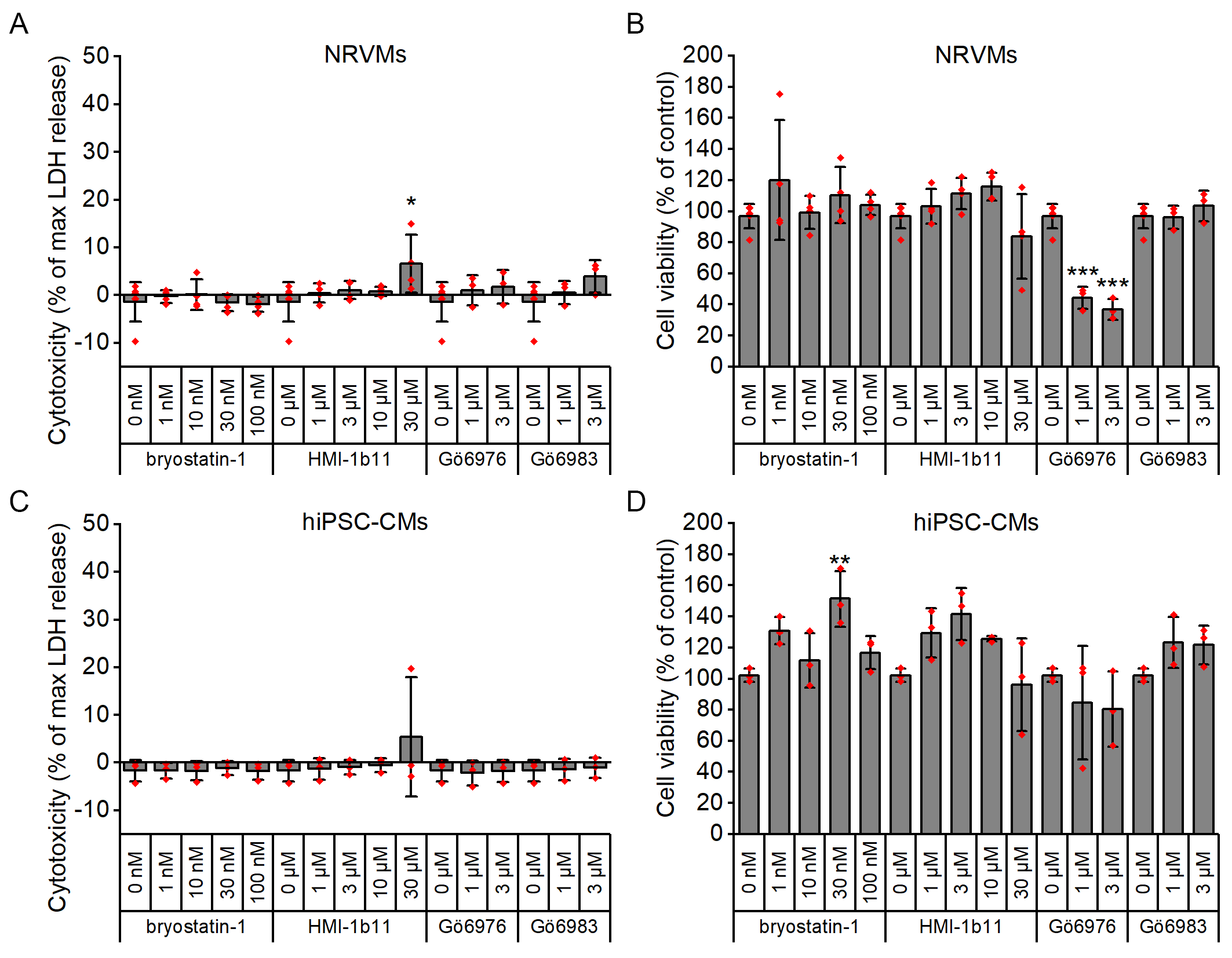

Supplement: Supplementary file 2 [file image1.tif]

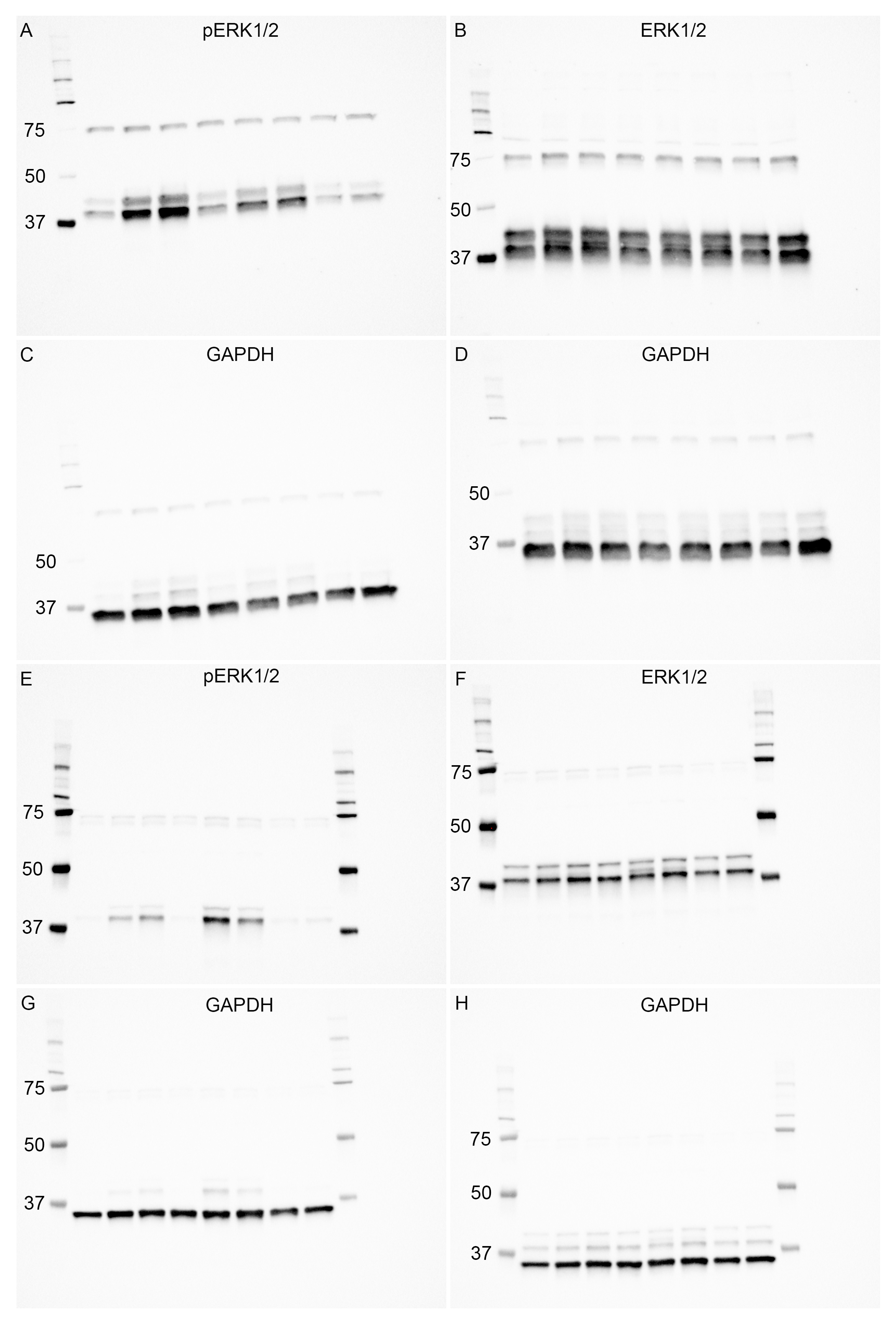

Supplement: Supplementary file 3 [file image2.tif]

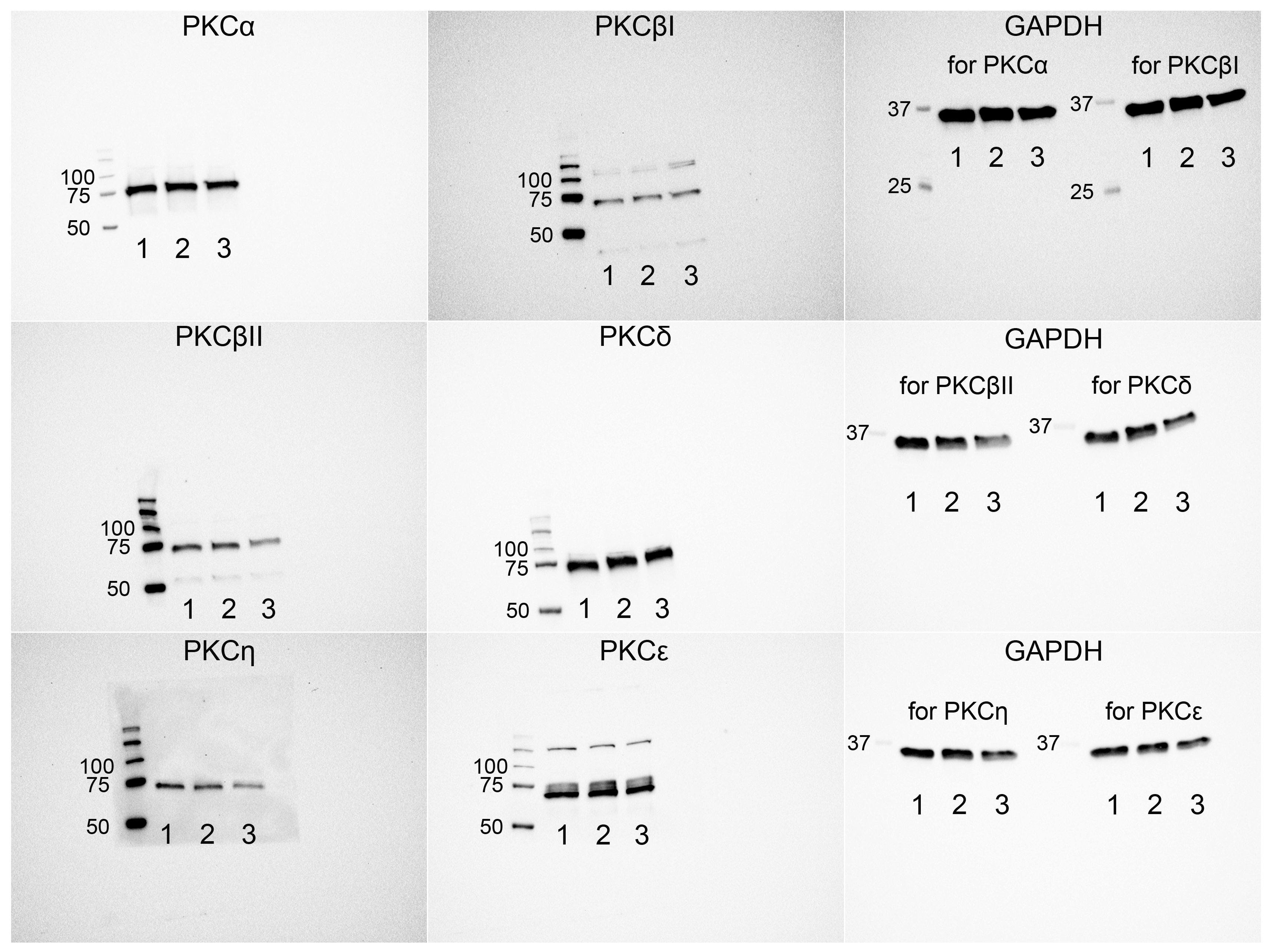

Supplement: Supplementary file 4 [file image3.tif]

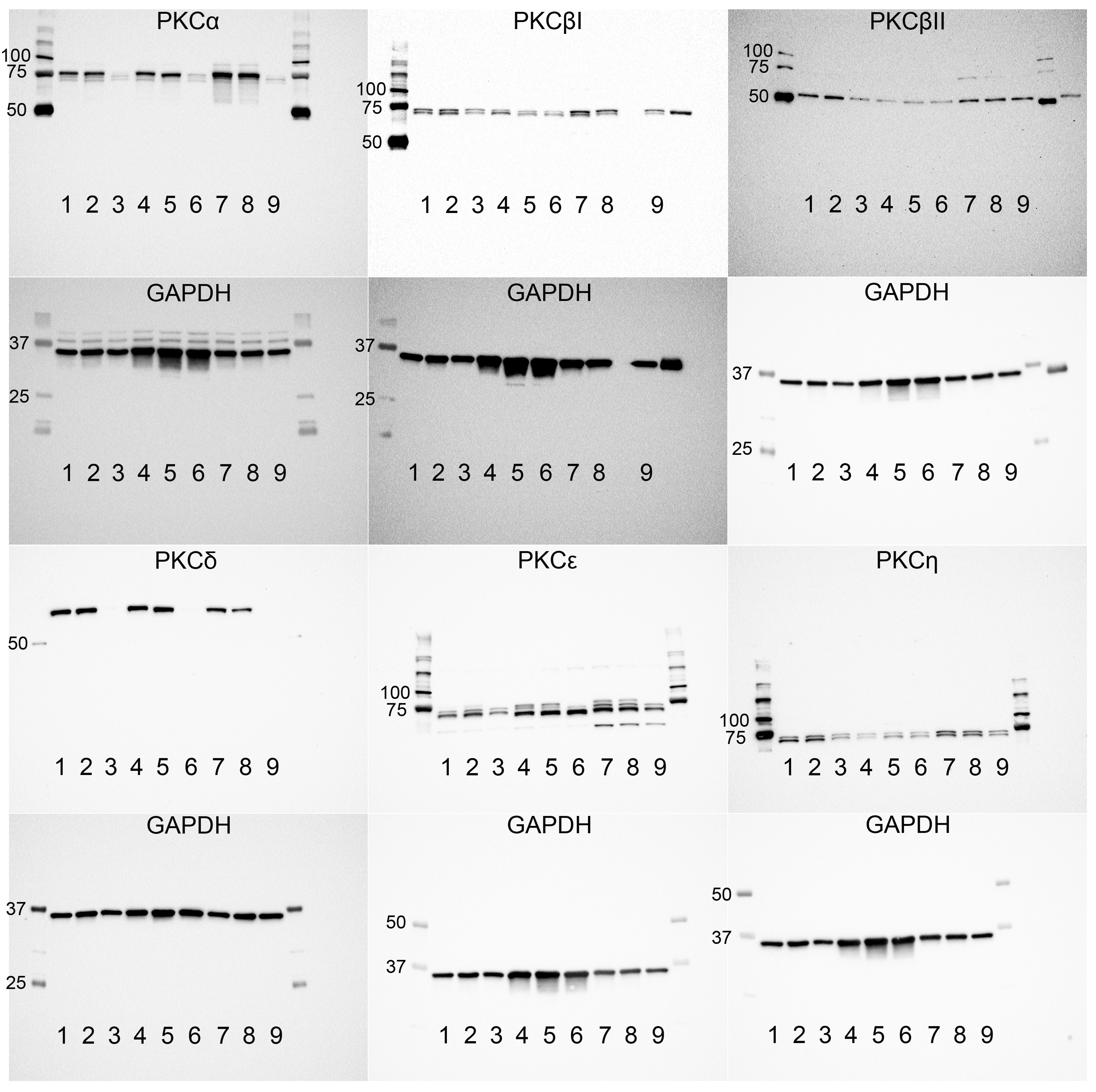

Supplement: Supplementary file 5 [file image4.tif]

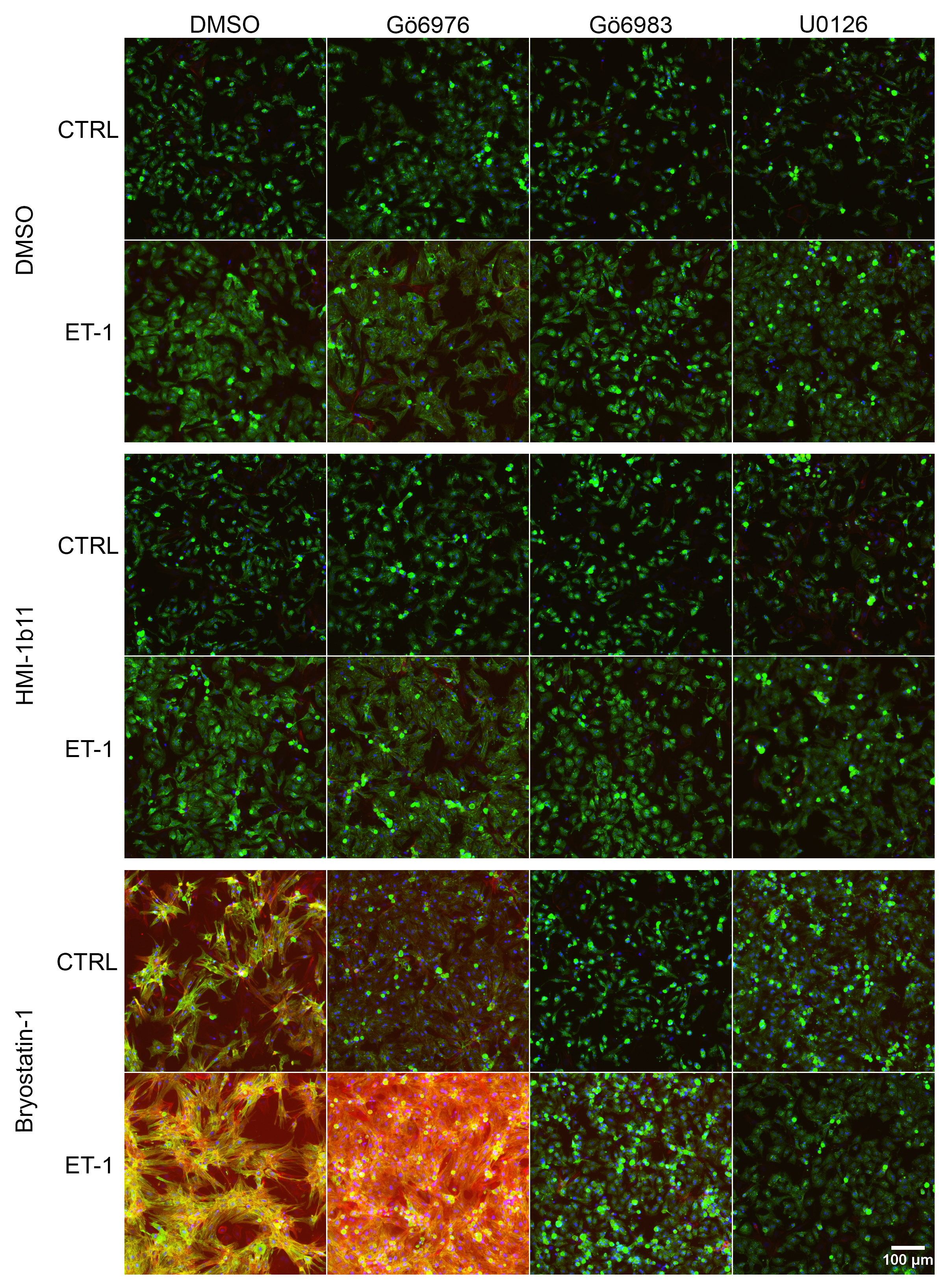

Supplement: Supplementary file 6 [file image5.tif]

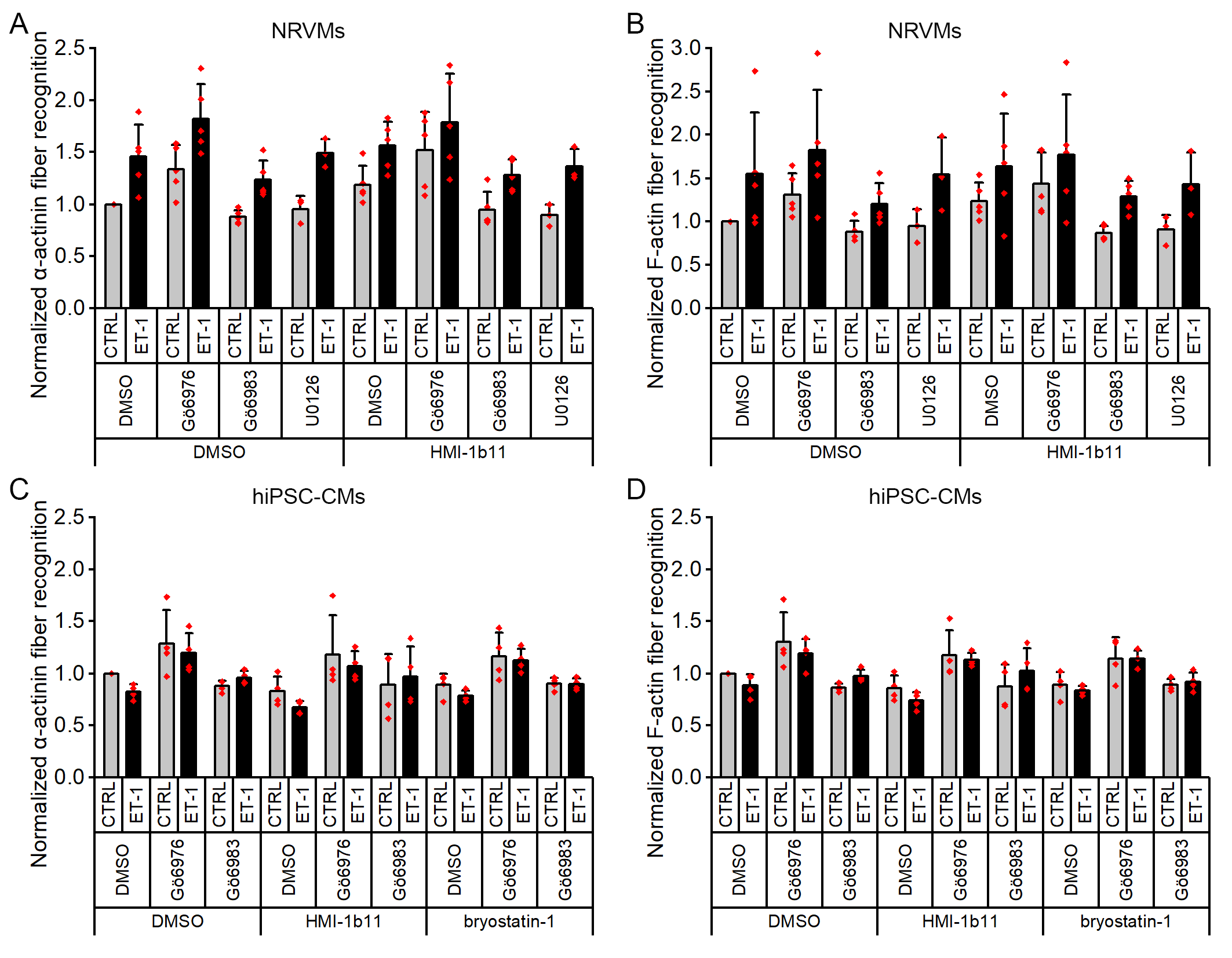

Supplement: Supplementary file 7 [file image6.tif]
